# Rapid detection of *Phytophthora cinnamomi* based on a new target gene *Pcinn13739*

**DOI:** 10.3389/fcimb.2022.923700

**Published:** 2022-08-25

**Authors:** Zhenpeng Chen, Binbin Jiao, Jing Zhou, Haibin He, Tingting Dai

**Affiliations:** ^1^ Co-Innovation Center for the Sustainable Forestry in Southern China, Nanjing Forestry University, Nanjing, China; ^2^ Inspection and Quarantine Technology Communication Department, Shanghai Customs College, Shanghai, China; ^3^ Technical Center for Animal, Plant and Food Inspection and Quarantine of Shanghai Customs, Shanghai, China

**Keywords:** *Phytophthora dieback*, *Phytophthora cinnamomi*, rapid diagnosis, recombinase polymerase amplification, lateral flow dipstick

## Abstract

*Phytophthora cinnamomi* causes crown and root wilting in more than 5,000 plant species and represents a significant threat to the health of natural ecosystems and horticultural crops. The early and accurate detection of *P. cinnamomi* is a fundamental step in disease prevention and appropriate management. In this study, based on public genomic sequence data and bioinformatic analysis of several *Phytophthora*, *Phytopythium*, and *Pythium* species, we have identified a new target gene, Pcinn13739; this allowed us to establish a recombinase polymerase amplification–lateral flow dipstick (RPA-LFD) assay for the detection of *P. cinnamomi*. *Pcinn13739*-RPA-LFD assay was highly specific to *P. cinnamomi*. Test results for 12 isolates of *P. cinnamomi* were positive, but negative for 50 isolates of 25 kinds of *Phytophthora* species, 13 isolates of 10 kinds of *Phytopythium* and *Pythium* species, 32 isolates of 26 kinds of fungi species, and 11 isolates of two kinds of *Bursaphelenchus* species. By detecting as little as 10 pg.µl^−1^ of genomic DNA from *P. cinnamomi* in a 50-µl reaction, the RPA-LFD assay was 100 times more sensitive than conventional PCR assays. By using RPA-LFD assay, *P. cinnamomi* was also detected on artificially inoculated fruit from *Malus pumila*, the leaves of *Rhododendron pulchrum*, the roots of sterile *Lupinus polyphyllus*, and the artificially inoculated soil. Results in this study indicated that this sensitive, specific, and rapid RPA-LFD assay has potentially significant applications to diagnosing *P. cinnamomi*, especially under time- and resource-limited conditions.

## Introduction

*Phytophthora cinnamomi* Rands is a genus in the Oomycetes, a Class in Phylum Pseudofungi within the Kingdom Chromista (Beakes et al., 2012). Oomycetes are a class of ubiquitous, filamentous microorganisms that include some of the biggest threats to global food security and natural ecosystems. *P. cinnamomi* has been ranked in the Top oomycete plant pathogens based on scientific and economic importance, which can infect more than 5,000 species of plants (Kamoun et al., 2015; Hardham and Blackman, 2018). This pathogen has caused significant economic losses in the agricultural, forestry, and horticultural activities of more than 76 countries. *P. cinnamomi* had catastrophic consequences on natural ecosystems and biodiversity (Rands, 1922; Zentmyer, 1980; Erwin and Ribeiro, 1996). As a soil-borne pathogen, *P. cinnamomi* can produce thick-walled oospores in host roots and soil (McCarren et al., 2005). This characteristic allows the pathogen to survive without causing obvious damage to the plants during the initial stages of infection, thus making this pathogen very difficult to manage (Dunstan et al., 2010). An increase in drought stress cycles has led to an exacerbation in the pathogenic effect of *P. cinnamomi* (Corcobado et al., 2014; Umami et al., 2021). Therefore, *P. cinnamomi* has become known as a “biological bulldozer” that represented a significant threat to plant species in temperate regions (Engelbrecht et al., 2021). With regards to the morphological comparison of *Phytophthora* with *Phytopythium* and *Pythium*, which also belong to oomycete, it has been established that the papillate sporangium of *Phytophthora* never shows internal proliferation. The combination of internal proliferation and papillation was unique to the sporangia of *Phytopythium* and some *Pythium* species. *Pythium* species have very large sporangia with a tapering neck rather than distinct papillae (De Cock et al., 2015). Previous research established maximum likelihood phylogenetic trees based on nuclear ribosomal DNA (*LSU* and *SSU*) and mitochondrial DNA cytochrome oxidase subunit I (*CO*X I), thus confirming that *Phytophthora*, *Phytopythium*, and *Pythium* belong to three distinct branches (De Cock et al., 2015).

Accurate and rapid detection played a fundamental role in the management of *P. cinnamomi*. Traditional assays for detecting *P. cinnamomi* included the isolation of samples from symptomatic plant tissues and baiting from soil (Erwin and Ribeiro, 1996). However, these methods required basic knowledge of cultural techniques and ideal laboratory conditions and involve long incubation and growth times. With the advent of molecular technology, several molecular assays have been designed to detect *P. cinnamomi*, namely, conventional polymerase chain reaction (PCR) (Kong et al., 2003), nested PCR (Williams et al., 2009), quantitative PCR (qPCR) (Eshraghi et al., 2011), Loop-mediated Isothermal Amplification (LAMP) (Dai et al., 2019), and recombinase polymerase amplification–lateral flow dipstick (RPA-LFD) (Dai et al., 2020).

The choice of the target for molecular detection was the first and most important step in the management of *P. cinnamomi*. Initially, Kong et al. (2003) designed specific PCR primers for detection based on the internal transcribed spacer (ITS) region. Williams et al. (2009) used nested multiplex PCR primers based on ITS to detect *P. cinnamomi* in the southwestern corner of Australia. Eshraghi et al. (2011) established a qPCR assay for detecting *P. cinnamomi* based on phosphate dikinase (*PDK*) gene. More recently, faster and easier isothermal assays have been developed, namely, LAMP (Notomi et al., 2000) and RPA. Dai et al. (2019) used whole genome sequencing to identify a new target gene in *P. cinnamomi* and then used the gene to develop a LAMP assay. The open reading frame (ORF) identification process used in this assay involved bioinformatics-based screening of all putative *P. cinnamomi* ORFs against eight *Phytopthora* databases (*P. capsica*, *P. infestans*, *P. nicotianae*, *P. ramorum*, *P. sojae*, *P. asiatica* (= *P. cinnamomi* var. *robiniae*), *P. parvispora* (= *P. cinnamomi* var. *parvispora*), and *P. vignae*. These species are closely related to *P. cinnamomi*. By combining sequence information from these species with those that were commonly encountered in the forestry environment, it will be helpful to search for target genes that are unique for *P. cinnamomi*. These genes will represent excellent candidates for application in rapid, field-based diagnostic assays that will be specific to *P. cinnamomi*.

Unlike the conventional PCR that requires denaturation, annealing, and extension and requires gel electrophoresis for imaging, RPA only requires 20 min of isothermal amplification at 37°C and 5 min of LFD to complete the visualization and detection (Daher et al., 2016). The RPA-LFD assays require a pair of forward primer and a 5′-biotin-labeled reverse primer and a special nfo probe with a fluorescein amidites (FAM) (carboxyfluorescein) antigen label at the 5′-end, a C3 spacer (amplification blocker) at the 3′-end, a tetrahydrofuran (THF) spacer (abasic site) in the middle. When the THF site pair and the template could match, the nfo enzyme was activated, the THF was cleaved, and the C3 spacer was excised from the probe. Thus, RPA contained both FAM and biotin, and the mouse anti-FAM antibody was enveloped with AuNPs. After diluted reaction products were added to the sample well, they were moved across the conjugate pad and binded to the anti-FAM AuNPs. The test line, which was enveloped with streptavidin, captures molecules with a biotin label when the amplification products went through. The control line, which was used to validate LFD detection, captured the anti-FAM antibody enveloped with AuNPs, because the anti-FAM antibody is from a mouse (Dai et al., 2019; Zhou et al., 2022).

In this study, public genomic sequence data and bioinformatic analysis of several *Phytophthora*, *Phytopythium*, and *Pythium* species were utilized for identifying the unique ORFs present in *P. cinnamomi*. These analyses identified a total of more than 1,000 specific target genes as the candidate genes for *P. cinnamomi*. Specific primers and probes were designed to amplify the novel target gene *Pcinn13739*. The specificity of the developed assay was evaluated by testing 50 isolates of *Phytophthora* species, six isolates of *Phytopythium* species, seven isolates of *Pythium* species, 32 isolates of fungal species, and 11 isolates of *Bursaphelenchus* species. Moreover, the sensitivity of the *Pcinn13739*-RPA-LFD assay was compared to a conventional PCR-based method.

## Materials and methods

### Maintenance of isolates and DNA extraction

The isolates of *Phytophthora*, *Phytopythium*, *Pythium*, *Bursaphelenchus*, and fungi used in this study were isolated from different host plants and regions (**Table 1**). *Phytophthora*, *Phytopythium*, and *Pythium* were cultured on 10% clarified V8 juice agar (cV8A). Isolates of fungi were grown on potato dextrose agar (PDA) at 25°C in the dark for 3–5 days. *B. xylophilus* and *B.mucronatus* were propagated using the mycelia of *Botrytis xylophilus* for one generation at 25°C for 4–5 days (Mamiya, 1975). The isolates used in this study are preserved in the Department of Forest Protection at Nanjing Forestry University in Nanjing, China. We used a DNAsecure Plant Kit (Tiangen Biotech, Beijing, China) to extract genomic DNA (gDNA) from all isolates. Extracted gDNAs were then quantified using a NanoDrop 2000c spectrophotometer (Thermo Fisher Scientific, MA, USA) and diluted accordingly. All DNA samples were stored at -20°C until use.

**Table 1:** Isolates used for specificity test of the RPA-LFD assay.

(sub) clade	Species	Isolate	Host/substrate	Location	RPA-LFD result^a^
7c	*P. cinnamomi*	ATCC 15400	*Cedrus deodara*	JS	+
		7308	*Castanopsis* sp.	Taiwan	+
		7514	*Castanopsis* sp.	Taiwan	+
		61J1	*Pieris* sp.	USA	+
		23B2	*Persea americana*	Puerto Rico	+
		CI	*Rhododendron pulchrum*	JS	+
		7574	*Pieris* sp.	USA	+
		7491	soil	USA	+
		7560	*Castanopsis* sp.	Taiwan	+
		CHAP1	*Cupressus funebris*	SX	+
		ACCC36284	soil	SX	+
		ZYM-1	*Rhododendron simsii*	SX	+
7c	*P. parvispora*	CBS132771	*Arbutus unedo*	Italy	−
		CBS132772	*Arbutus unedo*	Italy	−
7a	*P. cambivora*	CBS 248.60	*Castanea sativa*	USA	−
		Pc1	*Malus domestica Borkh*	SH	−
7a	*P. fragariae*	CBS209.46	*Fragaria × ananassa*	England, UK	−
7a	*P. fragariae* var. *rubi*	CBS 967.95	*Raspberry*	USA	−
7b	*P. melonis*	PMNJHG1	*Cucumis sativus*	JS	−
		PMNJHG2	*Cucumis sativus*	JS	−
		PMNJHG3	*Cucumis sativus*	JS	−
		PMNJDG1	*Benincasa hispida*	JS	−
		PMNJDG2	*Benincasa hispida*	JS	−
		PMNJDG3	*Benincasa hispida*	JS	−
		PMFJHL1	*Lagenaria siceraria*	FJ	−
		IMI 325917	*Cucumis* sp.	FJ	−
7b	*P. sojae*	P6497	*Glycine max*	USA	−
		R3	*Glycine max*	China	−
		R20	*Glycine max*	China	−
1a	*P. cactorum*	Pcac1	*Rosa chinensis*	FJ	−
		C1	*Malus pumila*	JS	−
		C2	*Malus pumila*	JS	−
		C3	*Rosa chinensis*	JS	−
1c	*P. infestans*	Pin1	*Solanum tuberosum*	FJ	−
		Pi2	*Solanum tuberosum*	YN	−
1	*P. nicotianae*	Pn1	*Nicotiana tabacum*	FJ	−
		Pn2	*Lycopersicum* sp.	JS	−
		Pn3	*Sophora sinensis*	JS	−
		Pn4	*Citrus* sp.	JS	−
		Pni1	*Nicotiana tabacum*	YN	−
2c	*P. capsici*	Pcap1	*Capsicum annuum*	JS	−
2c	*P. pini*	Ppini	*Rhododendron pulchrum*	JS	−
3	*P. ilicis*	CBS114348	*Ilex aquifolium*	Netherlands	−
4	*P. palmivora*	Ppa1	*Iridaceae*	YN	−
4	*P. quercetorum*	15C7	Soil	South Carolina, USA	−
4	*P. quercina*	CBS 789.95	*Quercus petraea*	Australia	−
4	*P. litchii*	Pli	*Litchi chinensis*	JS	−
5	*P. castaneae*	CBS587.85	Soil	Taiwan	−
6	*P. medicaginis*	ATCC 44390	*Medicago sativa*	USA	−
6	*P. megasperma*	CBS305.36	*Matthiola incana*	USA	−
6	*P. mississippiae*	57J3	*Irrigation water*	Mississippi, USA	−
8b	*P. brassicae*	CBS178.87	*Brassica* sp.	Canda	−
8	*P. hibernalis*	947	*Malus domestica Borkh*	SH	−
		CBS 132.23	*Unknown*	USA	−
8d	*P. syringae*	9099	*Malus domestica Borkh*	SH	−
		CBS 270.31	*Citrus sinesis*	United Kingdom	−
8c	*P. ramorum*	EU1 2275	*Quercus palustris*	United Kingdom	−
10	*P. boehmeriae*	Pb1	*Gossypium* sp.	JS	−
		PbI	*Boehmeria nivea*	JS	−
		Pb2	*Gossypium* sp.	JS	−
		Pb3	*B. nivea*	JS	−
		Pb4	*Gossypium* sp.	JS	−
Oomycete	*Phytopythium litorale*	PC-dj1	*Rhododendron simsii*	JS	−
		PC-dj2	*Rhododendron simsii*	JS	−
	*Ph. helicoides*	PH-C	*Rhododendron simsii*	JS	−
		PF-he2	*Rhododendron simsii*	JS	−
		PF-he3	*Rhododendron simsii*	JS	−
	*Ph. diclinum*	RS-di1	*Rhododendron simsii*	JS	−
	*Pythium aphanidermatum*	NT-ap1	*Nicotiana tabacum*	JS	−
	*Py. dissotocum*	RS-di2	*Rhododendron simsii*	JS	−
	*Py. spinosum*	OS-sp1	*Oryza sativa*	JS	−
	*Py. catenulatum*	RS-ca1	*Rhododendron simsii*	JS	−
	*Py. ultimum*	GM-ul1	*Glycine max*	JS	−
	*Py. torulosum*	RS-to1	*Rhododendron simsii*	JS	−
	*Py. plurisporium*	RS-pl1	*Rhododendron simsii*	JS	−
Fungi	*Fusarium acuminatum*	Fac1	*Rhizophora apiculata*	SC	−
	*F. asiaticum*	Fas1	*Triticum aestivum*	JS	−
	*F. avenaceum*	Fav1	*Glycine max*	JS	−
	*F. circinatum*	A045-1	*Pinus* sp.	SH	−
		A045-2	*Pinus* sp.	SH	−
		A045-3	*Pinus* sp.	SH	−
		A045-4	*Pinus* sp.	SH	−
	*F. fujikuroi*	Ffu1	*Oryza sativa*	JS	−
	*F. graminearum*	Fgr1	*Triticum aestivum*	JS	−
	*F. incarnatum*	IL3HQ	*Medicago sativa*	JS	−
	*F. lateritium*	Flat1	Soil	JS	−
	*F. moniforme*	Fmo1	*Oryza sativa*	JS	−
	*F. nivale*	Fniv	*Triticum aestivum*	JS	−
	*F. culmorum*	Fcu1	*Glycine max*	SC	−
	*F. commune*	Fco1	Soil	HLJ	−
	*F. equiseti*	Feq1	*Gossypium* sp.	JS	−
	*F. oxysporium*	Fox1	*Gossypium* sp.	JS	−
		Fox2	*Pinus* sp.	JS	−
	*F. proliferatum*	Fpr1	*Pinus* sp.	JS	−
		Fpr2	*Oryza sativa*	JS	−
	*F. solani*	Fso1	*Gossypium* sp.	JS	−
		Fso2	*Glycine max*	JS	−
	*Colletotrichum truncatum*	Ctr1	*Glycine max*	JS	−
	*C. glycines*	Cgl1	*Glycine max*	JS	−
	*C.orbiculare*	Cor1	*Citrullus lanatus*	JS	−
	*Verticilium dahliae*	Vda1	*Gossypium* sp.	JS	−
	*Rhizoctonia solani*	Rso1	*Gossypium* sp.	JS	−
	*Magnaporthe grisea*	Guy11	*Oryza sativa*	Japan	−
	*Alternaria alternata*	Aal1	Soil	JS	−
	*Tilletia indica*	Tin1	*Triticum aestivum*	JS	−
	*Diaporthe mahothocarpus*	DT1	*Kerria japonica*	JS	−
	*Botryosphaeria dothidea*	Bci1	*Koelreuteria paniculata*	JS	−
*Bursaphelenchus*	*Bursaphelenchus xylophilus*	JS-1	*Pinus thunbergii*	JS	−
		AH15	*Pinus thunbergii*	AH	−
		AH17	*Pinus thunbergii*	AH	−
		LN11	*Pinus thunbergii*	LN	−
		LN13	*Pinus thunbergii*	LN	−
		GX04	*Pinus thunbergii*	GX	−
		GX11	*Pinus thunbergii*	GX	−
	*B.mucronatus*	HUBM-1	*Pinus massoniana*	HB	−
		HUBM-10	*Pinus massoniana*	HB	−
		BM-9	*Pinus massoniana*	AH	−
		SHX-3	*Pinus massoniana*	SX	−

Abbreviation for Chinese provinces and municipalities: AH, Anhui; FJ, Fujian; GX, Guangxi; HB, Hubei; HLJ, Heilongjiang; JS, Jiangsu; LN, Liaoning; SC, Sichuan; SH, Shanghai; SX, Shanxi; YN, Yunnan.

a The positive and negative RPA-LFD results are represented by “+” and “−,” respectively.

### Primers and probe design

The annotated genomic sequence of *P. cinnamomi* at the website (https://mycocosm.jgi.doe.gov/Phyci1/Phyci1.home.html) was retrieved. Bioinformatics technology has developed significantly over recent years and, thus, a greater number of whole genome sequences were available for *Phytophthora*, *Phytopythium*, and *Pythium*, along with other fungal species that caused soil-borne disease. All 26,131 gene sequences of *P. cinnamomi* were used as queries to perform BLAST searches against the genomic sequences of 35 *Phytophthora* species derived from the National Center for Biotechnology Information (NCBI) database (E-value cutoff: 1 × 10^5^) (**Supplementary Table 1**). A total of more than 1,000 *P. cinnamomi* genes did not share any sequence similarity with any other species of *Phytophthora* and were used as queries to blast against the NCBI nucletotide database to further exclude genes showing similarity with the other nine *Pythium* species, five *Fusarium* species, and *Phytopythium* vexans. Ten genes (*Pcinn13739*, *Pcinn1025*, *Pcinn16994*, *Pcinn18321*, *Pcinn10079*, *Pcinn137495*, *Pcinn14424*, *Pcinn11754*, *Pcinn1601*, and *Pcinn17552*) were randomly selected as specific targets to *P. cinnamomi* for further conventional PCR primer design (**Supplementary Table 2**). PCR method was used to verify the specificity of these 10 target genes. These genes also did BLAST through the website (https://fungidb.org/fungidb/app/search/transcript/UnifiedBlast) to make sure its specificity and then used MEGA 7.0.20 to establish phylogenetic relationships. Then, we synthesized RPA primers and probes directly with a GenDx ERA Kit (GenDx Biotech, Suzhou, China) based on the specific gene in accordance with the manufacturer’s guidelines (**Supplementary Figure S1**). All primers and probes were synthesized by GenScript (Nanjing, China).

### Conventional PCR assay and nested PCR assay

Conventional PCR amplifications were performed in 50-µl reactions. Each reaction included 25 µl of Primer STAR Max Premix (2 × Takara), 21 µl of nuclease-free H_2_O, 2 µl of purified gDNA (100 ng), and 1 µl of each forward and reverse primer (10 µM). Thermal cycling began at 94°C for 3 min, followed by 33 cycles of 94°C for 30 s, 60°C for 30 s, and 72°C for 45 s; this was followed by 10 min at 72°C for 10 min. PCR was performed on Applied Biosystems Veriti Dx 96-Well Thermal Cycler (Thermo Fisher Scientific, Massachusetts, USA). Each set of reactions included a non-template control (NTC) to eliminate false positives. Following amplification, PCR products were detected on a 1.5% agarose gel, separated by electrophoresis at 130 V for approximately 25 min, and then visualized with a UV transilluminator. For nested PCR assays, we initially amplified the target gene of *P. cinnamomi* using specific primers (*Pcinn13739*-nest-F and *Pcinn13739*-nest-R). The thermal cycling program was the same as for conventional PCR (**Table 1**). For the second round of amplification, the PCR products arose from the first round, and then we added 2 μl of PCR products to each reaction and performed amplifications with *Pcinn13739*-F and *Pcinn13739*-R. All PCR and nested PCR reactions were repeated at least three times.

### RPA-LFD assays

**Figure 1:**
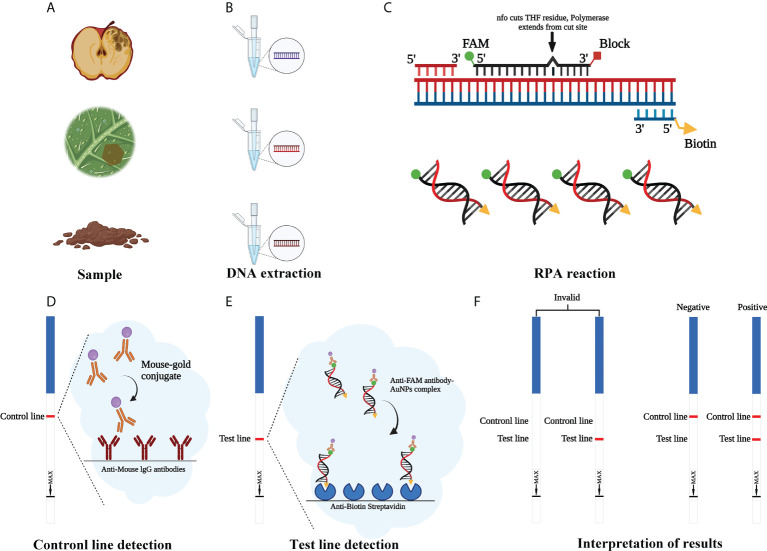
Schematic illustration of the RPA-LFD assay used to detect *Phytophthora cinnamomi.* **(A)** Collect the samples. **(B)** Genomic DNA (gDNA) is extracted from samples using the NaOH method. **(C)** Recombinase polymerase amplification (RPA). **(D)** Mouse antibody-gold conjugate binds to immobilized anti-mouse IgG antibodies. **(E)** The sample enters testing line and anti-FAM antibody-AuNPs complex binds to immobilized anti-biotin streptavidin. **(F)** Invalid result: no visible dipstick in control line despite the test line. Positive result: one visible dipstick each in control line and test line. Negative result: one visible dipstick in control line. Created with https://app.biorender.com/biorender-templates.

RPA assays were performed with a GenDx ERA kit (GenDx Biotech, Suzhou, China) in accordance with the manufacturer’s guidelines. Then, 5 µl of amplification product was diluted 60-fold with nuclease-free H_2_O. LFD was inserted into a centrifuge tube containing the diluted reaction products for 7–10 min to allow analysis of the assay results. When a red band appeared on both the control line (upper line) and test line (lower line) of the LFD, the result was considered to be positive. If it appears only on the control line and not on the test line, the LFD result was negative. Also, if it appears not on the control line, the LFD result was invalid (**Figure 1**). Tests were repeated at least three times. After visualization, LFDs were photographed using an EOS-750D camera (Canon, Tokyo, Japan).

### Evaluations of specificity between conventional PCR and RPA-LFD

The specificity of RPA-LFD system was evaluated with gDNA of 50 isolates of 25 kinds of *Phytophthora* species, 13 isolates of 10 kinds of *Phytopythium *and *Pythium* species, 32 isolates of 26 kinds of fungi (**Table 1**). A purified isolate of gDNA (100 ng) was used as a template for both assays. A positive control (*P. cinnamomi isolate 23B1*, 100 ng as a template) and a control (NTC, ddH_2_O as a template) were included in each set of reactions. All template concentrations were evaluated twice in each assay.

### Comparative evaluations of sensitivity between conventional PCR, nested PCR, and RPA-LFD

In a comparative evaluation of assay sensitivity, nine serial dilutions of gDNA from *P. cinnamomi* (100 ng, 10 ng, 1 ng, 100 pg, 10 pg, 1 pg, 100 fg, 10 fg, and 1 fg) were used in 50-µl reactions as templates for conventional PCR, nested PCR, and RPA-LFD assays. Each set of reactions included an NTC (ddH_2_O as template). All template concentrations were evaluated twice in each assay.

The use of conventional PCR and RPA-LFD assays to detect *P. cinnamomi* in artificially inoculated fruit from Malus pumila “FuJi,” the leaves of *Rhododendron pulchrum* Sweet, and the roots of sterile *Lupinus polyphyllus* Lindl

Prior to inoculation, the fruit of *M. pumila* “FuJi” and the leaves from *R. pulchrum* were washed with distilled water, immersed in 70% ethanol for 10 s, and then washed with sterilized distilled water. The fruit of *M. pumila* “FuJi” was then punctured with a sterile inoculation needle (to a depth of approximately 1 cm). The leaves of *R. pulchrum* were punctured with a sterile inoculation needle. A 5-day-old cV8A plug (5 × 5 mm) containing mycelium colonized by *P. cinnamomi* was then placed into the wound sites on six replicate fruits and leaves. Inoculated on the leaves of *R. pulchrum* using the mycelium with other species such as *Phytopythium litorale*, *Ph. helicoides*, and *Phytophthora pini* were also as controls. Non-inoculated control samples were treated with sterile cV8A plugs as negative controls. All samples were then moisturized and stored in a dark incubator at a temperature of 25°C. Three days later, gDNAs were extracted from decayed sections infested with *P. cinnamomi* using the NaOH method (Wang et al., 1993). The gDNA extractions were used as detection templates for conventional PCR and RPA-LFD. This experiment was carried out twice. Purified gDNA (100 ng) of *P. cinnamomi* was used as the template in positive control reactions and each set of reactions included NTC.

Seeds from *Lupinus polyphyllus* were first soaked in sterile water for 2 h, drained on filter paper, soaked in 70% ethanol for 30 s, rinsed three times in sterile water, soaked in 30% H_2_O_2_ for 10 min, rinsed four to five times in sterile water, and finally drained on sterile gauze for subsequent use. Sterile seeds were placed in Woody Plant Medium (WPM) (McCown and Loyd, 1981), with five seeds placed on each tray on the center line, covered in each WPM medium, sealed with a film cover, and then incubated in an incubator (temperature: 26°C; daylight: 16 h) for 5 days. When the seeds had grown to a root size of 1–2 cm, we selected healthy sterile seedlings for inoculation with *P. cinnamomi*. Then, we used the NaOH method (Wang et al., 1993) to extract gDNA from the decayed sections infested with *P. cinnamomi* and used the gDNA as amplification templates for conventional PCR and RPA-LFD. This experiment was carried out twice. Purified gDNA (100 ng) of *P. cinnamomi* was used as a template in positive control reactions. Each set of reactions included an NTC.

### Detection of *P. cinnamomi* in artificially inoculated soil

Ten agar plugs (2 × 2 mm^2^) of each *P. cinnamomi* isolate were transferred into 10 ml of 10% V8 juice to produce mycelial mats. After 3 days, the V8 was replaced with sterile water. To stimulate sporangial and zoospore production, three to five drops of soil extract solution (soil collected from healthy fields, immersed in sterile water, and filtered) were added to each plate. Then, 100 g of dried, sterile, and organic soil was inoculated with 50 ml of zoospore suspension (10^6^ zoospore/ml); then, 1-year-old *R. pulchrum* seedlings were planted in the soil. A portion of non-inoculated sterilized soil was used as a negative control. Ten days later, 100 mg of inoculated and non-inoculated soil was collected, and DNA was extracted using the E.Z.N.A. ^®^ Soil DNA Kit (Omega BIO TEK, Norcross, GA, USA). DNA templates were then used for RPA-LFD detection. This experiment was repeated three times.

## Results

### A novel PCR assay using *Pcinn13739* as a target gene exhibited good specificity

**Figure 2:**
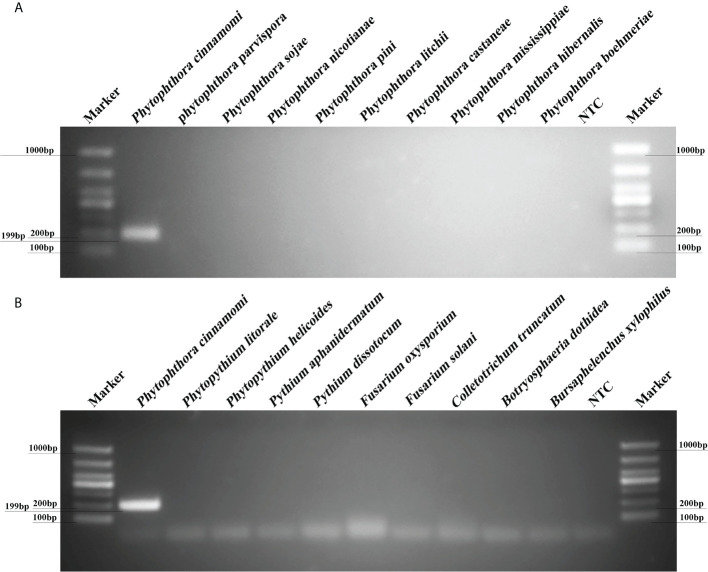
A new target gene (*Pcinn13739*) was found to be specific for the detection of *Phytophthora cinnamomi*. **(A)** Evaluation of specificity for a PCR assay based on a novel target (*Pcinn13739*) in *Phytophthora* species. Amplicons that were approximately 199 bp in size were detected by PCR in reactions containing gDNA from *P. cinnamomi* isolates. No PCR amplicons were observed for other *Phytophthora* species. **(B)** A 199 bp band was detected in *P. cinnamomi* but not in other *Phytopythium*, *Pythium*, fungi, *Bursaphelenchus*; similarly, no bands were detected in the negative controls NTC, negative control; Marker DL1000 (Takara Shuzo, Shiga, Japan). Each group of experiments was repeated in triplicate.

PCR reactions containing gDNAs from 12 isolates of *P. cinnamomi* isolates and 106 isolates of other *Phytophthora* species collected from different regions were used to test reaction specificity based on 10 target sequences. The *Pcinn13739-*PCR primers were designed to amplify specific target genes and were found to specific for *P. cinnamomi* (**Supplementary Table 2**). PCR amplicons that were approximately 199 bp in size were detected in PCR reactions containing gDNAs from *P. cinnamomi* isolates (**Figure 2**). No PCR amplicons were observed in reactions involving the isolates of 50 other *Phytophthora* species, six *Phytopythium* species, seven *Pythium* species, 32 fungal species, and 11 *Bursaphelenchus* species, including the closely related genera *P. asiatica* (= *P. cinnamomi* var. *robiniae*), *P. parvispora* (= *P. cinnamomi* var. *parvispora*), and *P. vignae*; similarly, no PCR amplicons were produced in the NTCs. In contrast, other primers, namely, *Pcinn16994*-F and *Pcinn16994*-R (based on *Pcinn16994*), *Pcinn18321*-F and *Pcinn18321*-R (based on *Pcinn18321*), *Pcinn10079*-F and *Pcinn10079*-R (based on *Pcinn10079*), *Pcinn1025*-F and *Pcinn1025*-R (based on *Pcinn1025*), *Pcinn11754*-F and *Pcinn11754*-R (based on *Pcinn11754*), *Pcinn1601*-F and *Pcinn1601*-R (based on *Pcinn1601*), *Pcinn137495*-F and *Pcinn137495*-R (based on *Pcinn137495*), *Pcinn14424*-F and *Pcinn14424*-R (based on *Pcinn14424*), and *Pcinn17552*-F and *Pcinn17552*-R (based on *Pcinn17552*) were used to test all experimental isolates. Other nine targets were not specific to *P. cinnamomi* (**Supplementary Figures S2**-**S6**). Phylogenetic relationships displayed that *Pcinn13739* was also a specific gene (**Supplementary Figure S7**). PCR amplicons were produced for the remaining nine genes, except for *Pcinn13739*, in reactions containing the gDNAs from other fungal or oomycete species, thus indicating that there was a lack of specificity for detecting *P. cinnamomi* DNA.

### Specificity of the *Pcinn13739-*RPA-LFD assay

**Figure 3:**
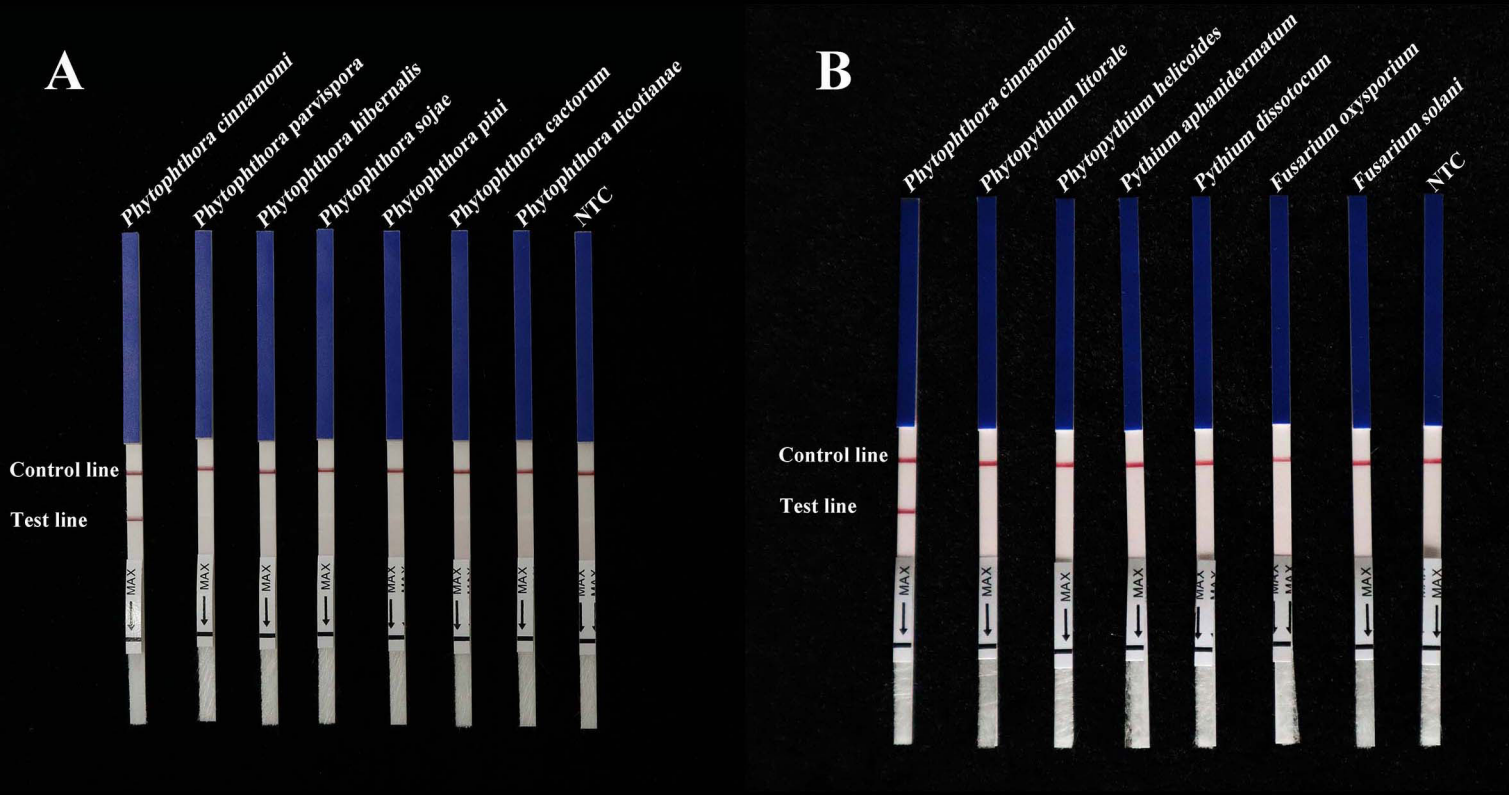
RPA-LFD assays were used to specifically detect *P. cinnamomi*. **(A)** All dipsticks yielded visible control lines, indicating validation tests. No test lines were detected in those featuring gDNA (100 ng) from other *Phytophthora* species or in NTCs. **(B)** Using the RPA-LFD assay, all dipsticks yielded visible control lines, indicating valid tests. No test line was detected for the gDNA (100 ng) of *Phytopythium* species, *Pythium* species, fungal species, *Bursaphelenchus* species, or in the NTCs. NTC, negative control. Experiments were repeated three times; all replicates showed the same results.

A specific pair of primers (*Pcinn13739*-RPA-F and *Pcinn13739*-RPA-R) was designed to exclusively and consistently amplify *P. cinnamomi* isolates by RPA-LFD assays (**Table 1**). The 5′-end of the reverse primer was labeled with biotin. A *Pcinn13739*-probe was also designed in accordance with the instructions provided with the GenDx ERA Kit (GenDx Biotech, Suzhou, China). In the RPA-LFD assay, the specificity of *Pcinn13739*-RPA-F and *Pcinn1373*9-RPA-R was verified using a variety of different species (50 isolates of *Phytophthora* spp., 13 isolates of *Phytopythium* and *Pythium* spp., 32 isolates of fungi, and 11 isolates of *Bursaphelenchus* spp. All dipsticks yielded visible control lines, indicating that the tests were valid. No test lines were detected in gDNAs (100 ng) from other species (*Phytophthora*, *Phytopythium*, *Pythium*, fungi, and *Bursaphelenchus*) or NTCs (**Figure 3**). Consistent results were obtained for the RPA-LFD assay in three biological replicates.

### Sensitivity of conventional PCR, nested PCR, and RPA-LFD assays

**Figure 4:**
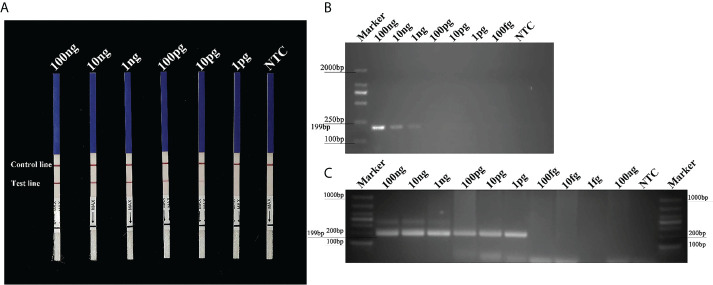
Sensitivity of the *Pcinn13739*-RPA-LFD, conventional PCR and nested PCR assays for *P. cinnamomi*. **(A)** All dipsticks yielded visible control lines, indicating that the test was valid. Test lines were visible on LFDs of RPA reactions (50 µl) containing at least 0.01 ng (10 pg) of *P. cinnamomi* gDNA. **(B)** In the conventional PCR assay, the detection threshold was 1 ng. No PCR amplicons were detected in reactions containing DNA template at 0.1 ng or lower. NTC, negative control. Marker DL2000 (Takara Shuzo, Shiga, Japan). **(C)** Nested PCR assay could detect 1 pg gDNA of *P. cinnamomi*, yielding positive detection results. NTC, negative control. Marker DL1000 (Takara Shuzo, Shiga, Japan). Experiments were repeated three times; all replicates showed the same results.

The *Pcinn13739*-RPA-LFD assays were 100-fold more sensitive than the conventional PCR assay for the detection of gDNA from *P. cinnamomi*. Sensitivity evaluation showed that RPA- LFDs had a visible control line. Test lines were visible on LFDs from RPA reactions (50 µl) containing at least 0.01 ng (10 pg) of *P. cinnamomi* gDNA (**Figure 4A**). No test lines were observed on those containing 1 pg of gDNA or the NTCs (**Figure 4A**). These results were consistent across three experimental replications. In the PCR assay, approximately 199-bp-long PCR amplicons were detected in reactions containing gDNA of *P. cinnamomi* isolates; no PCR amplicons were detected in reactions containing a concentration of DNA template that was 0.1 ng or lower (**Figure 4B**). The nested PCR assay detected 1 pg of *P. cinnamomi* gDNA and yielded positive detection results (**Figure 4C**). The sensitivity of nested PCR (1 pg) assay for the detection of *P. cinnamomi* was 10-fold higher than that of RPA-LFD (10 pg) and 1,000-fold higher than conventional PCR (1 ng). However, the time required for RPA-LFD (0.5 h) was only one-sixth of that required for nested PCR (3 h) and one-third of that for conventional PCR (1.5 h).

### Detection of *P. cinnamomi* by conventional PCR and RPA-LFD assays in artificially inoculated samples

**Figure 5:**
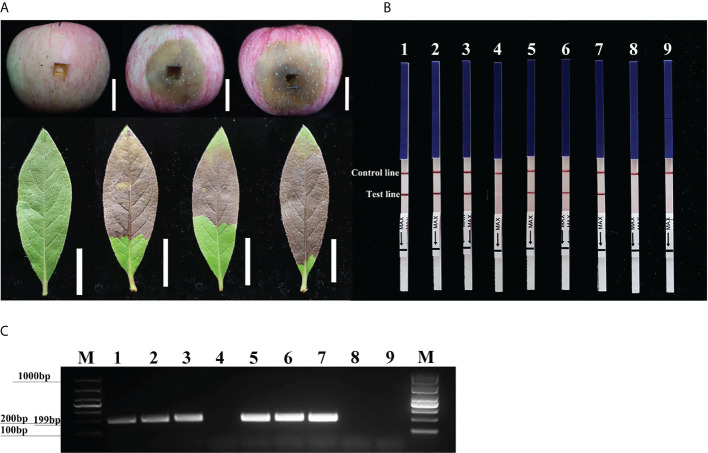
Detection of *Phytophthora cinnamomi* in artificially inoculated fruit from *Malus pumila* “FuJi” Mill. and the leaves of *Rhododendron pulchrum* Sweet using *Pcinn13739* RPA-LFD and conventional PCR assays. **(A)** 1–2, artificially inoculated *M. pumila* “FuJi.” 3, a healthy apple as a negative control. 4–6, artificially inoculated *R. pulchrum* leaves. 7, a healthy leave as a negative control. The white scale bar represents 2cm. **(B)** The detection of artificially inoculated samples by the RPA-LFD assay. 1–2, artificially inoculated *M. pumila* “FuJi.” 3, a non-inoculated apple as a negative control. 4–6, artificially inoculated *R. pulchrum* leaves. 7, non-inoculated leave as a negative control. NTC, ddH_2_O as template. Genomic DNA (100 ng) of *P. cinnamomi* was used as a template in positive control (PTC). All dipsticks yielded visible control lines, indicating valid tests. **(C)** Detection of gDNA extracted from artificially inoculated samples by conventional PCR assay. 1–2, artificially inoculated *M. pumila* “FuJi.” 3, non-inoculated apple as a negative control. 4–6, artificially inoculated *R. pulchrum* leaves. 7, non-inoculated leave as a negative control. Negative control (NTC), ddH_2_O as template. M, Marker DL1000 (Takara Shuzo, Shiga, Japan). Genomic DNA (100 ng) of *P. cinnamomi* was used as a template in positive control (PTC). Each set of experiments was repeated in triplicate with the same results.

Three days after inoculation with mycelium of *P. cinnamomi*, we observed typical symptoms at the inoculation sites on the fruit of *M. pumila* “FuJi” and the leaves of *R. pulchrum*; however, no such symptoms were evident on the non-inoculated negative controls (**Figure 5A**). gDNAs were extracted from the fruit of *M. pumila* “FuJi” and the leaves of *R. pulchrum* inoculated with *P. cinnamomi* to act as templates for assays. Red bands were evident on both the control and test lines of the LFDs, indicating positive test results. Correspondingly, two symptomatic fruits (1–2) and three symptomatic leaves (4–6) had positive results in the RPA-LFD assay, whereas the asymptomatic ones (3 and 7) and the NTCs (ddH_2_O as template) had negative results (**Figure 5B**). *P. cinnamomi* was recovered from symptomatic fruits but not from asymptomatic ones or the negative control. The conventional PCR assay also detected *P. cinnamomi* in the artificially inoculated samples (**Figure 5C**). PCR amplicons that were approximately 199 bp in size were detected in PCR reactions containing gDNAs from inoculated samples (1–2, 4–6), whereas no PCR amplicons were showed in the asymptomatic ones (3 and 7) and the NTCs (ddH_2_O as template). Each set of experiments was repeated three times, and the same results were obtained each time.

**Figure 6:**
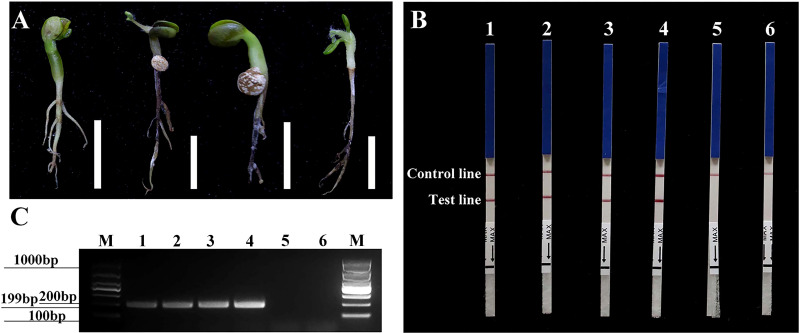
Detection of *Phytophthora cinnamomi* in the artificially inoculated roots of sterile *Lupinus polyphyllus* Lindl. using *Pcinn13739* RPA-LFD and conventional PCR assays. **(A)** 1–3, roots of artificially inoculated sterile *L. polyphyllus*. 4, non-inoculated as a control. The white scale bar represents 1 cm. **(B)** Detection of artificially inoculated samples by the RPA-LFD assay. 1–3, roots of artificially inoculated sterile *L. polyphyllus*. 4, non-inoculated as a control. Genomic DNA (100 ng) of *P. cinnamomi* was used as a template in positive control (PTC), negative control (NTC). All dipsticks yielded visible control lines, indicating valid tests. **(C)** Detection of artificially inoculated samples by conventional PCR assay. 1–3, roots of artificially inoculated sterile *L. polyphyllus*. 4, non-inoculated as a control. M, Marker DL1000 (Takara Shuzo, Shiga, Japan). Genomic DNA (100 ng) of *P. cinnamomi* was used as a template in positive control (PTC), negative control (NTC). Each group of experiments was repeated in triplicate and yielded similar results.

Typical symptoms of brown rot were observed in all roots of artificially inoculated *L. polyphyllus* samples (1–3) but not in the non-inoculated control *L. polyphylla* (4) (**Figure 6A**). In the *Pcinn13739*-RPA-LFD assay, all dipsticks had visible control lines. Test lines were visible on three dipsticks with total DNAs extracted from inoculated roots, whereas no test lines were observed on those from non-inoculated root and NTC (**Figure 6B**). PCR amplicons that were approximately 199 bp in size were detected in PCR reactions containing gDNAs from inoculated roots (1–3), whereas no PCR amplicons were showed in the asymptomatic one (4) and the NTC (ddH_2_O as template) **(** **Figure 6C**). Each group of experiments were repeated three times.

### Validation of the assays with artificially inoculated soil

**Figure 7:**
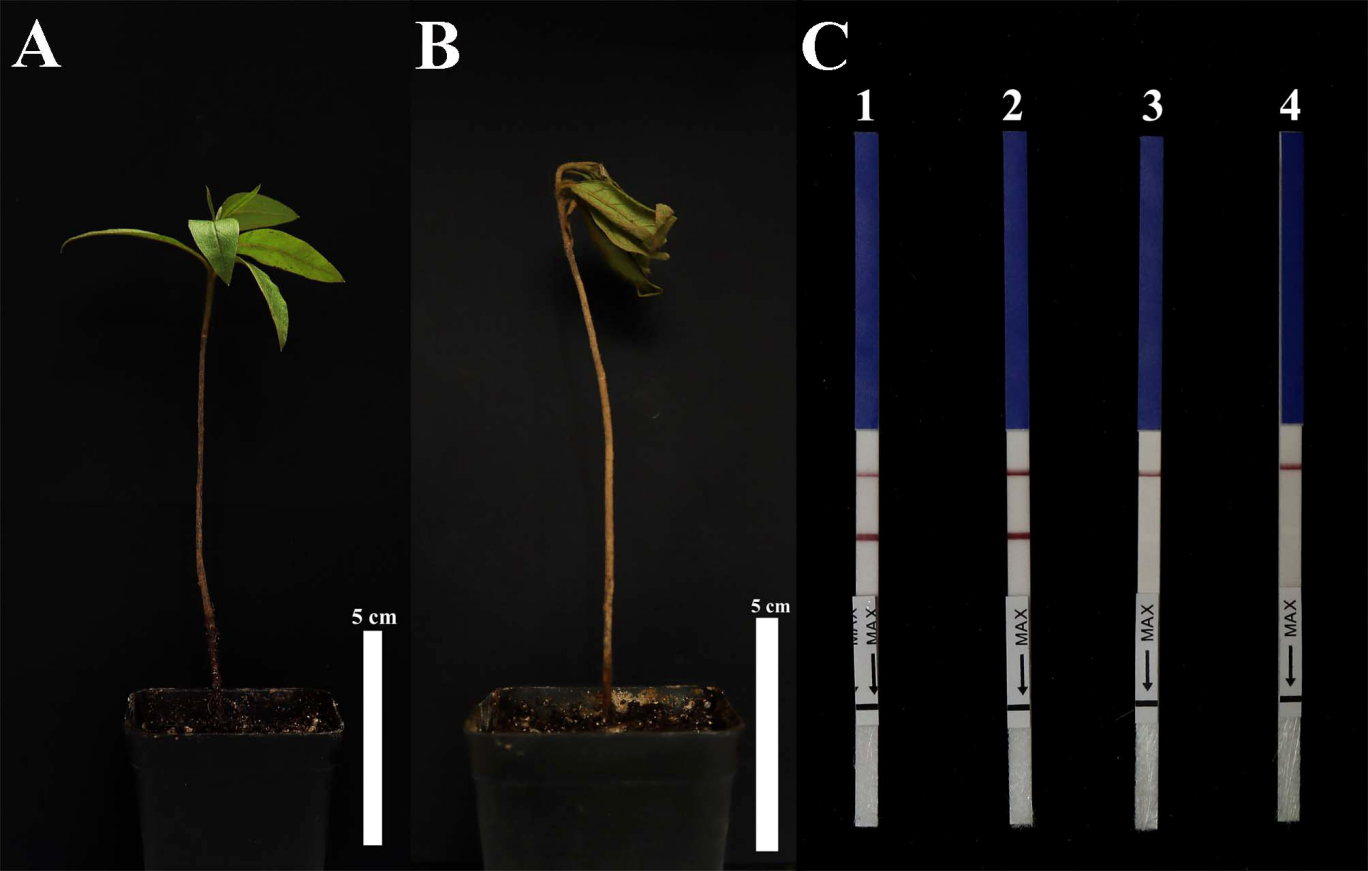
Detection of *Phytophthora cinnamomi* in artificially inoculated soil. **(A)** *R. pulchrum* was grown in non-inoculated soil for 10 days. **(B)** *R. pulchrum* was grown in artificially inoculated soil for 10 days. **(C)** Detection of artificially inoculated soil by RPA-LFD assay. 1, positive control. 2, artificially inoculated soil. 3, non-inoculated soil. 4, negative control. Data is representative of three replicate experiments.

Ten days after planting in soil containing zoospore suspension, *R. pulchrum* seedlings had developed disease symptoms, whereas the controls had no symptom (**Figures 7A, B** **)**. In total, 100 mg of inoculated and non-inoculated soil was collected, and DNA was extracted using the E.Z.N.A. ^®^ Soil DNA Kit. Then, we used the DNA as templates for RPA-LFD detection. gDNA (100 ng µl^-1^) purified from *P. cinnamomi* isolates was used as a positive control, whereas ddH_2_O was used as an NTC. In the RPA-LFD assay, all dipsticks showed a visible control line. Test lines were also visible on dipsticks containing DNA extracted from artificially *P. cinnamomi–*inoculated soils and the positive control, However, no test lines were observed from non-inoculated soil or the NTC (**Figure 7C**). Identical results were obtained from three repeats of this experiment.

## Discussion

*P. cinnamomi* has led to significant declines of the agricultural livestock ecosystem in the Iberian Peninsula (Rodriguez-Romero et al., 2021) and the production of highbush blueberry in Germany (Nechwatal and Jung, 2021) and also led to root and stem rot in *Vaccinium corymbosum* L. (Lan et al., 2016a) and *Castanea mollissima* (Chinese Chestnut) (Lan et al., 2016b). In addition, *P. cinnamomi* has had severe impacts on kiwifruit production in China (Bi et al., 2019) and has seriously affected the growth and yield of *Carya cathayensis* in Linan County, China (Tong et al., 2022). Due to the serious economic implications associated with *P. cinnamomi*, it was vital to develop a reliable and accurate diagnostic tool. To implement timely disease management and avoid unnecessary costs due to misdiagnosis and delayed diagnosis, accurate detection of *P. cinnamomi* was a prerequisite for effective management and successful elimination of pathogens. However, molecular detection methods, such as conventional PCR (Kong et al., 2003), nested PCR (Williams et al., 2009), and LAMP (Dai et al., 2019), were time-consuming and require personnel with professional knowledge, thus making it very difficult to detect *P. cinnamomi* on a wide scale.

The detection method of the RPA-LFD assay was judged by the test line and the control line which could not detect by gel except basic RPA method. So, we could not use a similar pattern of the figure to show the sensitivity either dipstick or gel or both. However, the three methods were used for detecting *P. cinnamomi *based on the target *Pcinn13739*, which have different sensitivity. The sensitivity of the RPA-LFD assay (10 pg) for detecting *P. cinnamomi* was 100-fold higher than conventional PCR (1 ng) assay. RPA-LFD testing was advantageous, because it was time-saving, sensitive, and required no specialist operators. Dai et al. (2019) developed LAMP assays for detecting *P. cinnamomi* in soil and targeted a new target gene (*Pcinn100006*) that had been identified from genomic sequencing data. Over recent years, there have been many reports of pathogens causing crown and root rot in *Rhododendron pulchrum*, including *P. pini* (Xu et al., 2021), *Phytopythium littorale* (Li et al., 2021), and *Phytopythium helicoides* (Chen et al., 2021) except *P. cinnamomi*. Therefore, more isolates need to be used to verify the specificity of the RPA-LFD assay, especially those causing diseases in the same host. The number of species (118 isolates from 64 species) tested in this study is greater than the numbers tested in previous studies [76 isolates from 38 species (Dai et al., 2020), 50 isolates from 31 species (Dai et al., 2019), 53 isolates from 22 species (Engelbrecht et al., 2013), 74 isolates from 16 species (Williams et al., 2009), 37 isolates from 29 species (Kong et al., 2003)]. Based on these numbers, RPA primers and probe designed in RPA-LFD assay may be the most reliable for the specific detection of *P. cinnamomi*. Among the closely related genera, *P. parvispora* also gave negative results in *Pcinn13739 -*RPA-LFD assay. Screening for unique target genes was the most critical step for establishing fast and accurate molecular detection methods. ITS region, *Ypt1*, and β*-tubulin* loci have been used to identify *Phytophthora* (Kroon et al., 2004; Yang and Hong, 2018). However, due to the homology of these target genes, molecular detection method based on these genes often generate false positives (Manisha et al., 2017). Dai et al. (2019) developed a LAMP assay to detect *P. cinnamomi*. However, the LAMP-based assay requires at least four primer pairs and an amplification time of 1 h. Dai et al. (2020) designed a pair of specific primers to develop an RPA-LFD assay for the *P. cinnamomi* RxLR effector gene *PHYCI_587572*. However, effectors are secreted by the pathogen during its interaction with the host, interfering with the immune response of the plant and promoting infection (Jones and Dangl, 2006). Evolutionary analysis shows that RxLR and CRN effectors undergo genetic selection pressure (Jiang et al., 2008). Specific primers designed to target RxLR effector genes can result in the failure to amplify their target genes in an appropriate manner or generate false positives due to the presence of genetic selection pressure. With the rapid development of bioinformatics, the combination of bioinformatic analysis and comparative genomics to search for specific target genes has been widely applied, such as *Pectobacterium* species (Ahmed et al., 2018), *P. cinnamomi* (Dai et al., 2019), and *Schistosoma japonicum* (Guo et al., 2021).

In this study, based on public genomic sequence data and bioinformatic analysis of several *Phytophthora*, *Phytopythium*, and *Pythium* species, we have identified a new target gene, *Pcinn13739*, and used as a target gene to develop the conventional PCR and RPA-LFD assays. Unlike the conventional PCR that requires denaturation, annealing, and extension and requires gel electrophoresis for imaging, RPA only requires 20 min of isothermal amplification at 37°C and 5 min of LFD to complete the visualization and detection. There were no other report of isothermal amplification methods such as strand displacement isothermal amplification (SDA), helicase-dependent DNA isothermal amplification (HDA), and rolling loop isothermal amplification technology (RCA) for *P. cinnamomi* detection. CRISPR assay has become a very popular technology and has been deployed in a huge range of applications. The CRISPER/Cas12a assay is gradually being applied for the detection of pathogens (Chen et al., 2018; Li et al., 2018). In future, maybe a simple, rapid, sensitive, and unaided eye visualization CRISPR-Cas12a detection system will be developed for molecular identification of *P. cinnamomi* without requiring technical expertise or ancillary equipment.

## Conclusions

A new target gene, *Pcinn13739*, was identified by aligning the whole-genome sequences of *P. cinnamomi* with other species of *Phytophthora*, *Phytopythium*, and *Pythium*. We designed specific primers (*Pcinn13739*-RPA-F and *Pcinn13739*-R) to this new target gene. The specificity of this target gene was then tested in various assays by testing 50 isolates of other *Phytophthora* species, six *Phytopythium* species, seven *Pythium* species, 32 fungal species, and *Bursaphelenchus* species. The *Pcinn13739-*RPA-LFD detection assay was shown to detect 10 pg of *P. cinnamomi* gDNA in a 50-µl reaction. This assay also detected *P. cinnamomi* in the artificially inoculated fruit of *M. pumila* “FuJi,” the leaves of *R. pulchrum*, the roots of sterile *L. polyphyllus*, and in artificially inoculated soil. The detection method that we have developed, based on the RPA assay with LFDs, can be visualized without the need for extensive equipment, thus facilitating the early detection and prediction of *P. cinnamomi* and serving as an early warning system.

## Data availability statement

The original contributions presented in the study are included in the article/**Supplementary Material**. Further inquiries can be directed to the corresponding author.

## Author contributions

ZC conceptualized and designed the research, analyzed the data, interpreted the results, performed the experiments, and wrote the manuscript. BJ, JZ, and HH participated in and discussed the experimental design. TD revised the manuscript and directed the project. All authors contributed to the article and approved the submitted version.

## Funding

This research was supported by the National Key R&D Program of China (Reference: 2021YFD1400100 and 2021YFD1400103), the Natural Science Foundation of Jiangsu Province, China (BK 20191389), the Jiangsu University Natural Science Research Major Project (Reference: 21KJA220003), and the Qinglan Project of 2020 and the Priority Academic Program Development of Jiangsu Higher Education Institutions.

## Acknowledgments

The authors would like to thank Cuiping Wu at Jiangsu Costoms for providing the isolates and DNAs of *Phytophthora* species used in this study.

## Conflict of interest

The authors declare that the research was conducted in the absence of any commercial or financial relationships that could be construed as a potential conflict of interest.

## Publisher’s note

All claims expressed in this article are solely those of the authors and do not necessarily represent those of their affiliated organizations, or those of the publisher, the editors and the reviewers. Any product that may be evaluated in this article, or claim that may be made by its manufacturer, is not guaranteed or endorsed by the publisher.

## Supplementary material

Supplementary Figure 1 Sequence of the *Pcinn13739* gene of *Phytophthora cinnamomi*. The nucleotides targeted by the forward primer (*Pcinn13739*RPA-F), the *Pcinn13739*RPA-Probe, and the reverse primer (*Pcinn13739*RPA-R) in the novel recombinant polymerase amplification method are located below their respective arrows. The arrows indicate the direction of amplification.

Supplementary Figure 2 Evaluation of specificity of the PCR assay based on the target *Pcinn16994* and *Pcinn18321*. **(A)** Conventional PCR products amplified with primers *Pcinn16994*-F and *Pcinn16994*-R are detected by 2% agarose gel electrophoresis. **(B)** Conventional PCR products amplified with primers *Pcinn18321*-F and *Pcinn18321*-R are detected by 2% agarose gel electrophoresis. PCR amplicons were also detected in samples using gDNA from other *Phytophthora* species, indicating a lack of specificity in detection of *P. cinnamomi* DNA. *Pcinn16994* and *Pcinn18321* were found to be non-specific for detecting *P. cinnamomi*. Marker DL2000 (Takara Shuzo, Shiga, Japan). Negative control (NTC).

Supplementary Figure 3 Evaluation of specificity of the PCR assay based on the target *Pcinn10079* and *Pcinn1025*. **(A)** Conventional PCR products amplified with primers *Pcinn10079*-F and *Pcinn10079*-R are detected by 2% agarose gel electrophoresis. **(B)** Conventional PCR products amplified with primers *Pcinn1025*-F and *Pcinn1025*-R are detected by 2% agarose gel electrophoresis. PCR amplicons were also detected in samples using gDNA from other *Phytophthora* species, indicating a lack of specificity in detection of *P. cinnamomi* DNA. *Pcinn10079* and *Pcinn1025* were found to be non-specific for detecting *P. cinnamomi*. Marker DL1000 (Takara Shuzo, Shiga, Japan). Negative control (NTC).

Supplementary Figure 4 Evaluation of specificity of the PCR assay based on the target *Pcinn11754* and *Pcinn1601*. **(A)** Conventional PCR products amplified with primers *Pcinn11754*-F and *Pcinn11754*-R are detected by 2% agarose gel electrophoresis. **(B)** Conventional PCR products amplified with primers *Pcinn1601*-F and *Pcinn1601*-R are detected by 2% agarose gel electrophoresis. PCR amplicons were also detected in samples using gDNA from other *Phytophthora* species, indicating a lack of specificity in detection of *P. cinnamomi* DNA. *Pcinn11754* and *Pcinn1601* were found to be non-specific for detecting *P. cinnamomi*. Marker DL2000 (Takara Shuzo, Shiga, Japan). Negative control (NTC).

Supplementary Figure 5 Evaluation of specificity of the PCR assay based on the target *Pcinn137495* and *Pcinn14424*. **(A)** Conventional PCR products amplified with primers *Pcinn137495*-F and *Pcinn137495*-R are detected by 2% agarose gel electrophoresis. **(B)** Conventional PCR products amplified with primers *Pcinn14424*-F and *Pcinn14424*-R are detected by 2% agarose gel electrophoresis. PCR amplicons were also detected in samples using gDNA from other *Phytophthora* species, indicating a lack of specificity in detection of *P. cinnamomi* DNA. *Pcinn137495* and *Pcinn14424* were found to be non-specific for detecting *P. cinnamomi*. Marker DL2000 (Takara Shuzo, Shiga, Japan). Negative control (NTC).

Supplementary Figure 6 Evaluation of specificity of the PCR assay based on the target *Pcinn17552*. Conventional PCR products amplified with primers *Pcinn17552*-F and *Pcinn17552*-R are detected by 2% agarose gel electrophoresis. PCR amplicons were also detected in samples using gDNA from other *Phytophthora* species, indicating a lack of specificity in detection of *P. cinnamomi* DNA. *Pcinn17552* was found to be non-specific for detecting *P. cinnamomi*. Marker DL1000 (Takara Shuzo, Shiga, Japan). Negative control (NTC).

Supplementary Figure 7 Maximum parsimony phylogeny for *Pcinn13739*. The phylogenetic relationship of *Pcinn13739* derived from maximum-likelihood (ML) analysis. BLAST searches revealed *Pcinn13739* in different branches of a phylogenetic tree with nine closely related genes (https://fungidb.org/fungidb/app/search/transcript/UnifiedBlast).

Supplementary Table 1 Publicly available genome sequences of 34 Phytophthora species, eight Pythium species, five Fusarium species, and Phytopythium vexans.

Supplementary Table 2 Ten loci were randomly selected for screening by conventional PCR assays from more than 1,000 *Phytophthora cinnamomi*–specific putative genes.
