# Supplementary figures and images for: Rapid detection of Phytophthora cinnamomi based on a new target gene Pcinn13739
Source: Front Cell Infect Microbiol. 2022 Aug 25;12:923700. doi: 10.3389/fcimb.2022.923700 (PMC9452884; doi:10.3389/fcimb.2022.923700)

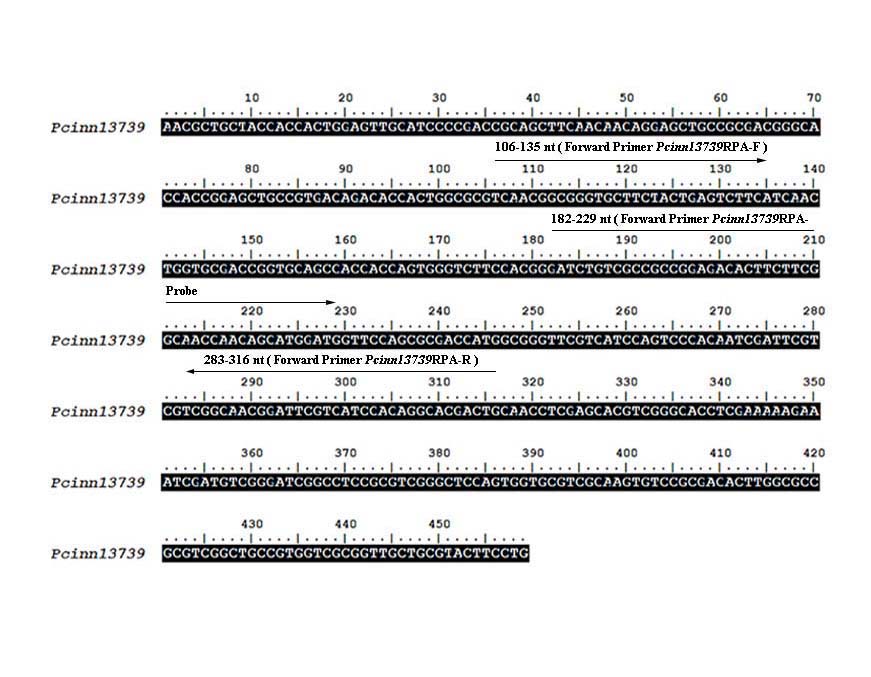

Supplement: Supplementary Figure 1 — Sequence of the Pcinn13739 gene of Phytophthora cinnamomi. The nucleotides targeted by the forward primer (Pcinn13739RPA-F), the Pcinn13739RPA-Probe, and the reverse primer (Pcinn13739RPA-R) in the novel recombinant polymerase amplification method are located below their respective arrows. The arrows indicate the direction of amplification. [file Image_1.jpeg]

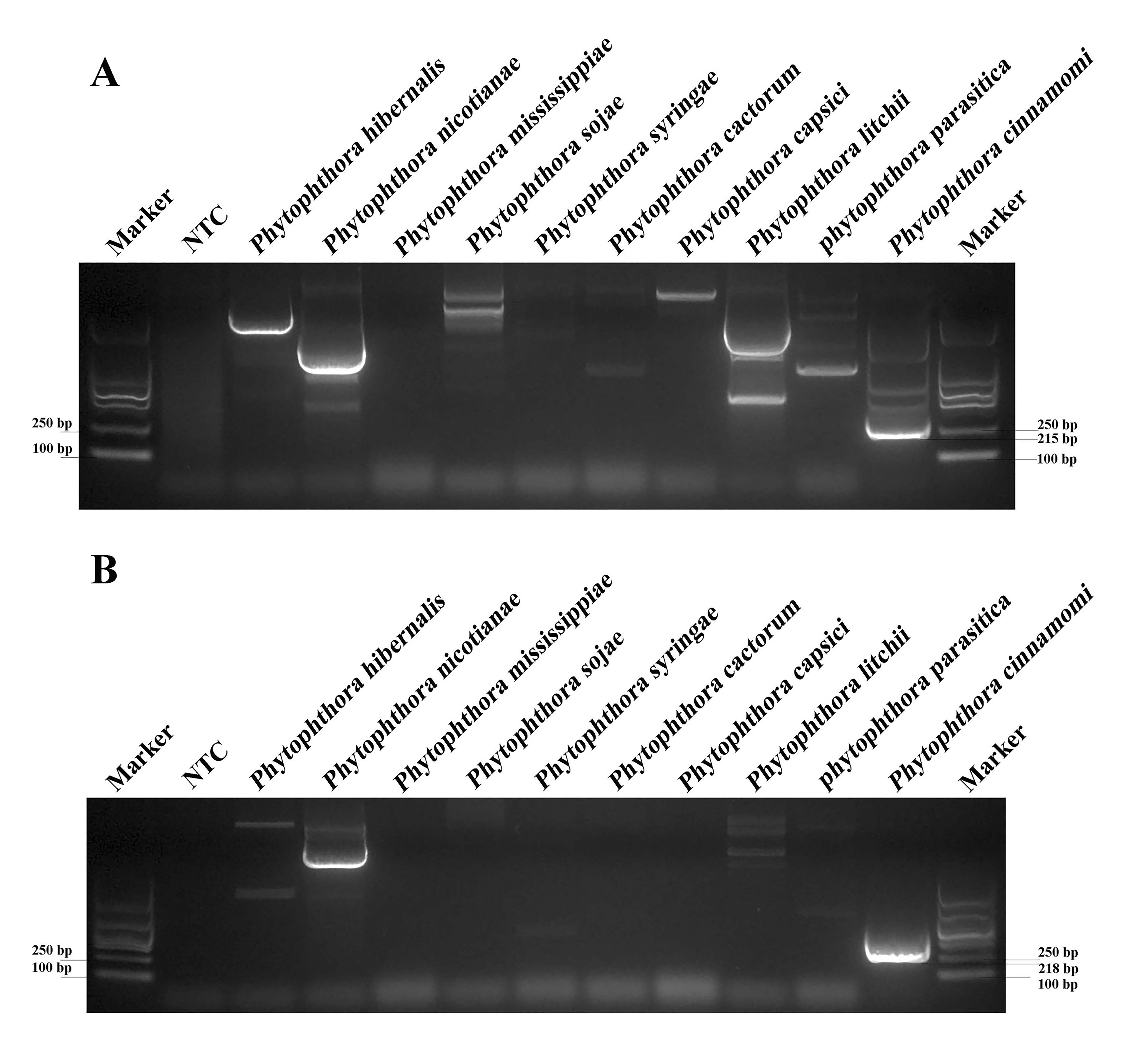

Supplement: Supplementary Figure 2 — Evaluation of specificity of the PCR assay based on the target Pcinn16994 and Pcinn18321. (A) Conventional PCR products amplified with primers Pcinn16994-F and Pcinn16994-R are detected by 2% agarose gel electrophoresis. (B) Conventional PCR products amplified with primers Pcinn18321-F and Pcinn18321-R are detected by 2% agarose gel electrophoresis. PCR amplicons were also detected in samples using gDNA from other Phytophthora species, indicating a lack of specificity in detection of P. cinnamomi DNA. Pcinn16994 and Pcinn18321 were found to be non-specific for detecting P. cinnamomi. Marker DL2000 (Takara Shuzo, Shiga, Japan). Negative control (NTC). [file Image_2.jpeg]

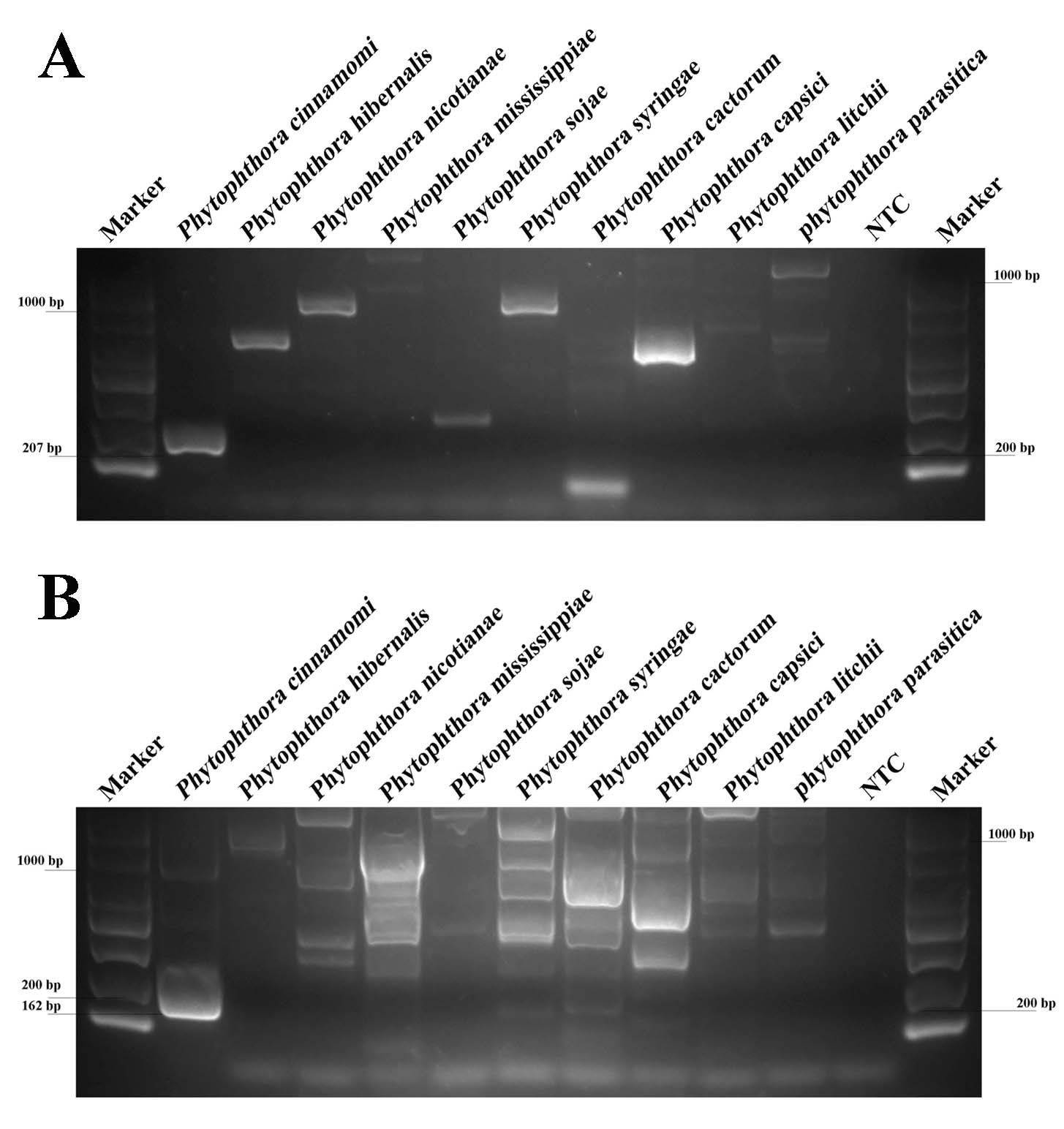

Supplement: Supplementary Figure 3 — Evaluation of specificity of the PCR assay based on the target Pcinn10079 and Pcinn1025. (A) Conventional PCR products amplified with primers Pcinn10079-F and Pcinn10079-R are detected by 2% agarose gel electrophoresis. (B) Conventional PCR products amplified with primers Pcinn1025-F and Pcinn1025-R are detected by 2% agarose gel electrophoresis. PCR amplicons were also detected in samples using gDNA from other Phytophthora species, indicating a lack of specificity in detection of P. cinnamomi DNA. Pcinn10079 and Pcinn1025 were found to be non-specific for detecting P. cinnamomi. Marker DL1000 (Takara Shuzo, Shiga, Japan). Negative control (NTC). [file Image_3.jpeg]

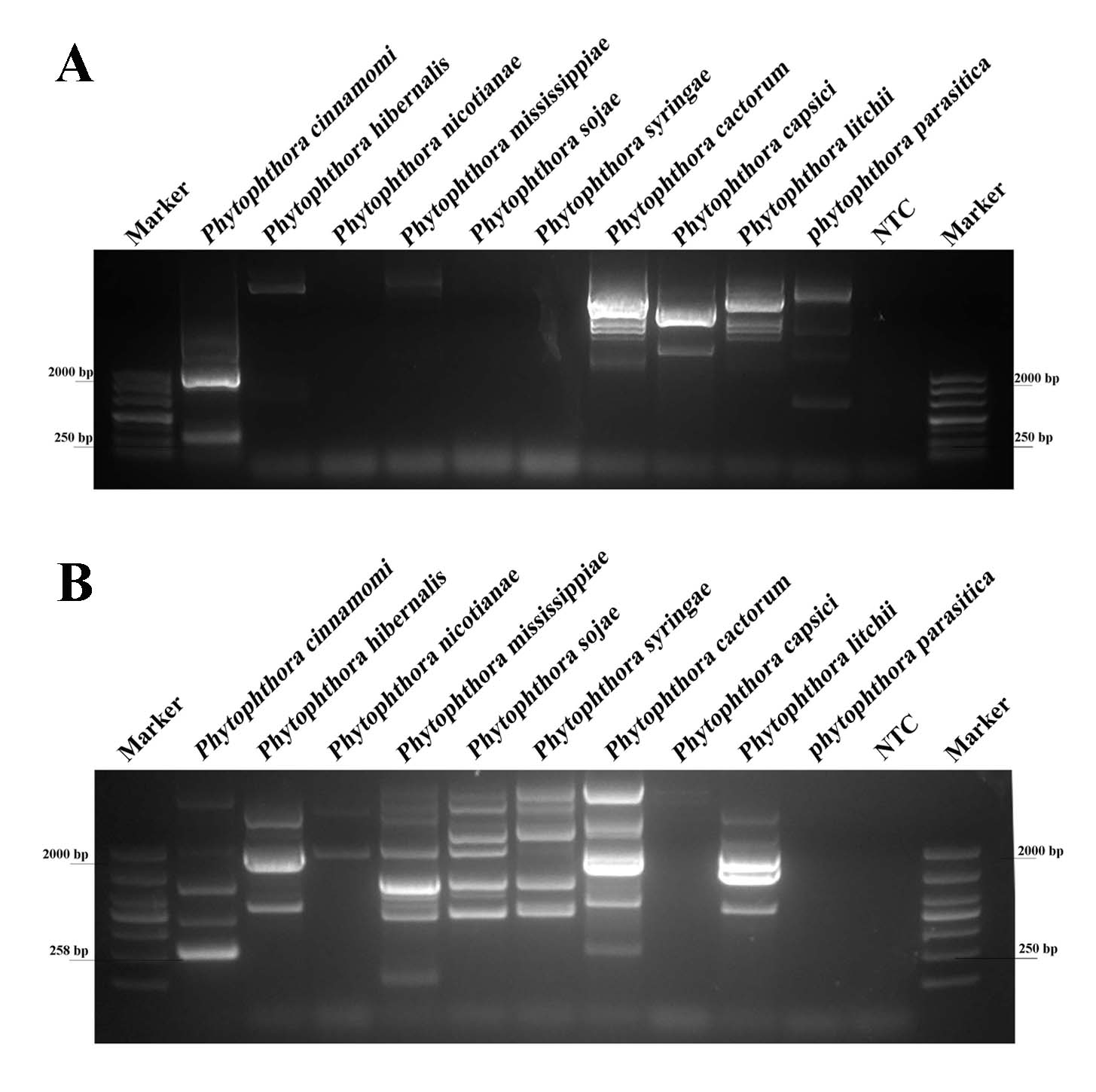

Supplement: Supplementary Figure 4 — Evaluation of specificity of the PCR assay based on the target Pcinn11754 and Pcinn1601. (A) Conventional PCR products amplified with primers Pcinn11754-F and Pcinn11754-R are detected by 2% agarose gel electrophoresis. (B) Conventional PCR products amplified with primers Pcinn1601-F and Pcinn1601-R are detected by 2% agarose gel electrophoresis. PCR amplicons were also detected in samples using gDNA from other Phytophthora species, indicating a lack of specificity in detection of P. cinnamomi DNA. Pcinn11754 and Pcinn1601 were found to be non-specific for detecting P. cinnamomi. Marker DL2000 (Takara Shuzo, Shiga, Japan). Negative control (NTC). [file Image_4.jpeg]

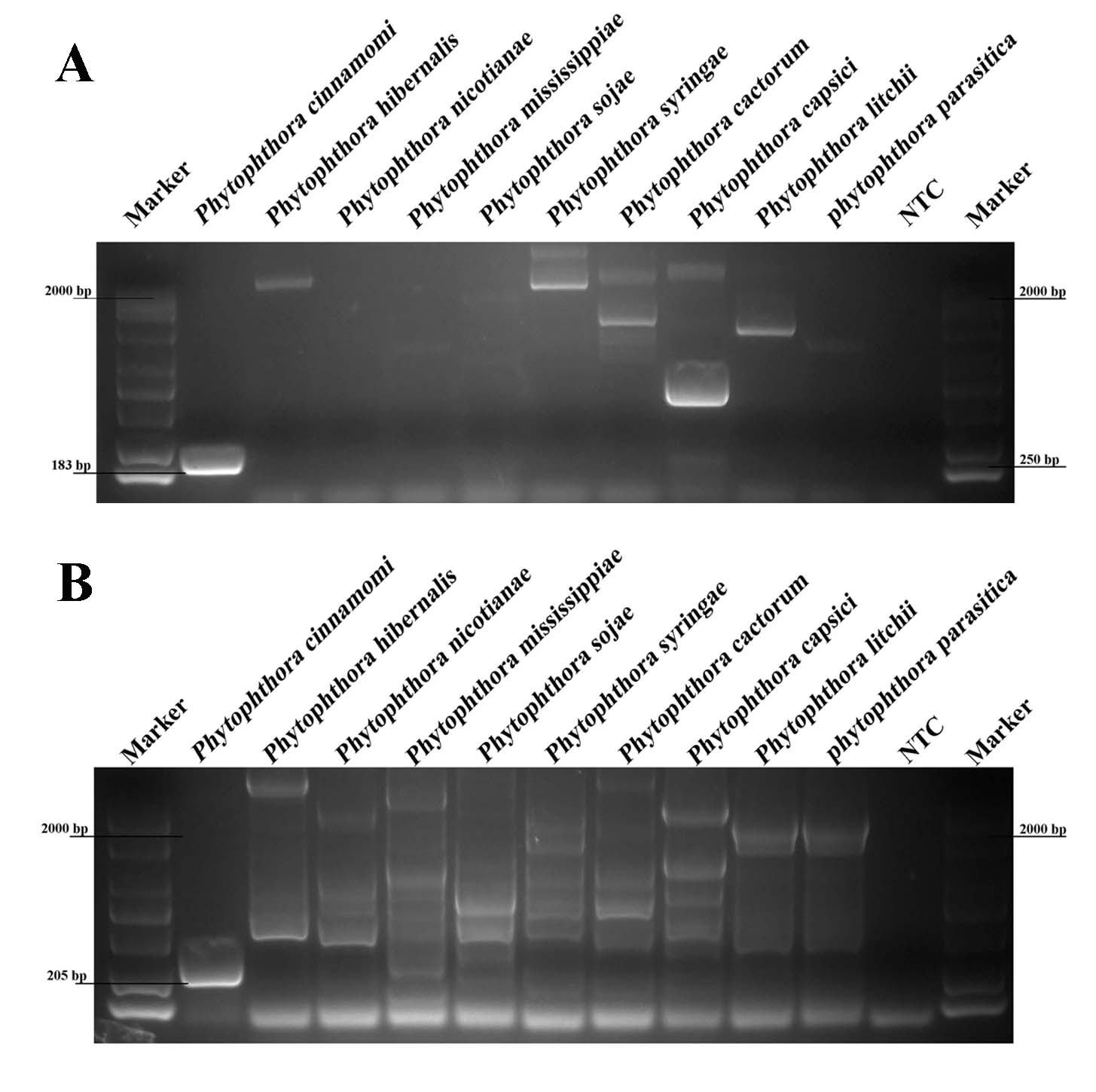

Supplement: Supplementary Figure 5 — Evaluation of specificity of the PCR assay based on the target Pcinn137495 and Pcinn14424. (A) Conventional PCR products amplified with primers Pcinn137495-F and Pcinn137495-R are detected by 2% agarose gel electrophoresis. (B) Conventional PCR products amplified with primers Pcinn14424-F and Pcinn14424-R are detected by 2% agarose gel electrophoresis. PCR amplicons were also detected in samples using gDNA from other Phytophthora species, indicating a lack of specificity in detection of P. cinnamomi DNA. Pcinn137495 and Pcinn14424 were found to be non-specific for detecting P. cinnamomi. Marker DL2000 (Takara Shuzo, Shiga, Japan). Negative control (NTC). [file Image_5.jpeg]

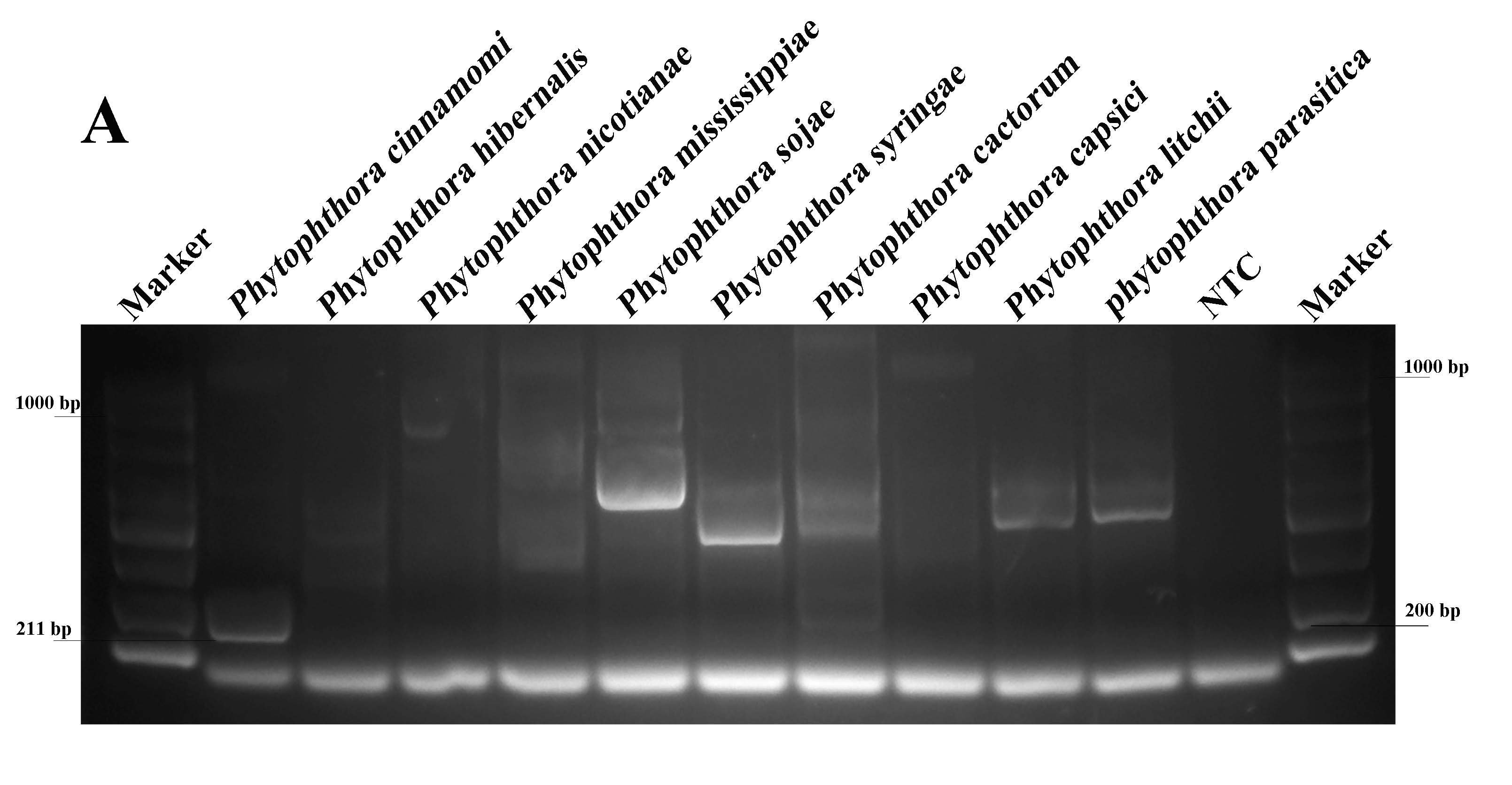

Supplement: Supplementary Figure 6 — Evaluation of specificity of the PCR assay based on the target Pcinn17552. Conventional PCR products amplified with primers Pcinn17552-F and Pcinn17552-R are detected by 2% agarose gel electrophoresis. PCR amplicons were also detected in samples using gDNA from other Phytophthora species, indicating a lack of specificity in detection of P. cinnamomi DNA. Pcinn17552 was found to be non-specific for detecting P. cinnamomi. Marker DL1000 (Takara Shuzo, Shiga, Japan). Negative control (NTC). [file Image_6.jpeg]

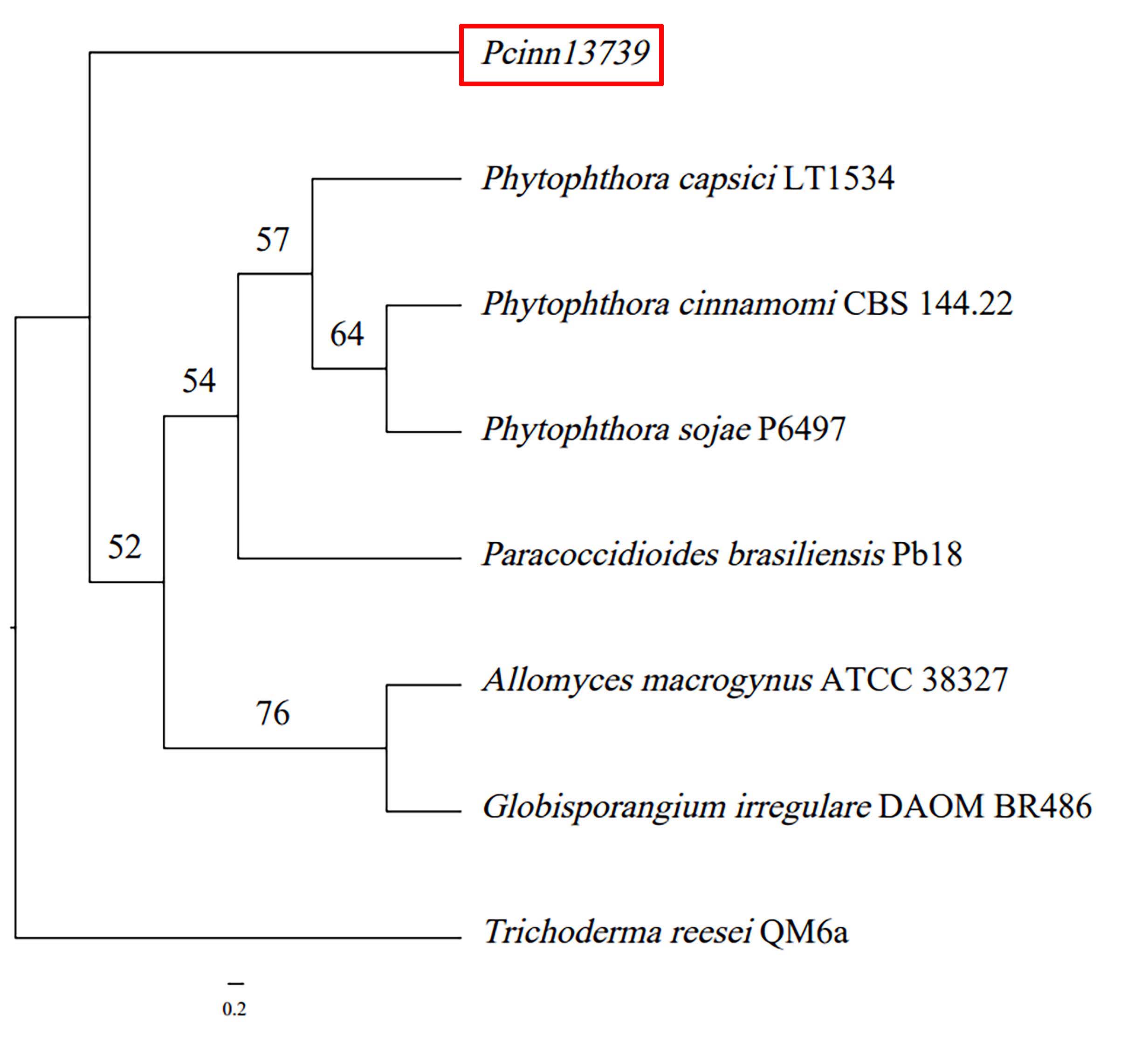

Supplement: Supplementary Figure 7 — Maximum parsimony phylogeny for Pcinn13739. The phylogenetic relationship of Pcinn13739 derived from maximum-likelihood (ML) analysis. BLAST searches revealed Pcinn13739 in different branches of a phylogenetic tree with nine closely related genes (https://fungidb.org/fungidb/app/search/transcript/UnifiedBlast). [file Image_7.jpeg]
