# Supplementary material for: Rapid detection of Phytophthora cinnamomi based on a new target gene Pcinn13739
Source: Front Cell Infect Microbiol. 2022 Aug 25;12:923700. doi: 10.3389/fcimb.2022.923700 (PMC9452884; doi:10.3389/fcimb.2022.923700)
Supplement: Supplementary Table 1 — Publicly available genome sequences of 34 Phytophthora species, eight Pythium species, five Fusarium species, and Phytopythium vexans. [file Table_1.doc]

| Table S1. Publicly available genome sequences of 34 Phytophthora species, 8 Pythium species, 5 Fusarium species, and Phytopythium vexans. | |
| --- | --- |
| **Species** | **Website** |
| Phytophthora aleatoria | *Phytophthora aleatoria* (ID 103392) - Genome - NCBI (nih.gov) |
| *P. betacei* | *Phytophthora betacei* (ID 88030) - Genome - NCBI (nih.gov) |
| *P. boehmeriae* | *Phytophthora boehmeriae* (ID 88770) - Genome - NCBI (nih.gov) |
| *P. cambivora* | *Phytophthora cambivora* (ID 16811) - Genome - NCBI (nih.gov) |
| *P. castanetorum* | *Phytophthora castanetorum* (ID 95290) - Genome - NCBI (nih.gov) |
| *P. chlamydospore* | *Phytophthora chlamydospora* (ID 88879) - Genome - NCBI (nih.gov) |
| *P. citricola* | *Phytophthora citricola* (ID 83150) - Genome - NCBI (nih.gov) |
| *P. constricta* | *Phytophthora constricta* (ID 88771) - Genome - NCBI (nih.gov) |
| *P.cryptogea* | *Phytophthora cryptogea* (ID 16812) - Genome - NCBI (nih.gov) |
| *P. fragariae* | *Phytophthora fragariae* (ID 31914) - Genome - NCBI (nih.gov) |
| *P. gonapodyides* | *Phytophthora gonapodyides* (ID 88881) - Genome - NCBI (nih.gov) |
| *P. hibernalis* | *Phytophthora hibernalis* (ID 88950) - Genome - NCBI (nih.gov) |
| *P. idaei* | *Phytophthora idaei* (ID 98698) - Genome - NCBI (nih.gov) |
| *P. kernoviae* | *Phytophthora kernoviae* (ID 15933) - Genome - NCBI (nih.gov) |
| *P. lateralis* | *Phytophthora lateralis* (ID 15280) - Genome - NCBI (nih.gov) |
| *P. litchi* | *Phytophthora litchii* (ID 65401) - Genome - NCBI (nih.gov) |
| *P. macrochlamydospora* | *Phytophthora macrochlamydospora* (ID 88773) - Genome - NCBI (nih.gov) |
| *P. megakarya* | *Phytophthora megakarya* (ID 55630) - Genome - NCBI (nih.gov) |
| *P. melonis* | *Phytophthora* melonis (ID 84282) - Genome - NCBI (nih.gov) |
| *P. multivora* | *Phytophthora multivora* (ID 40645) - Genome - NCBI (nih.gov) |
| *P. syringae* | *Phytophthora syringae* (ID 88951) - Genome - NCBI (nih.gov) |
| *P. tubulina* | *Phytophthora tubulina* (ID 95292) - Genome - NCBI (nih.gov) |
| *P. ohioensis* | *Phytophthora ohioensis* (nom. inval.) (ID 95291) - Genome - NCBI (nih.gov) |
| *P. palmivora* | *Phytophthora palmivora* (ID 66939) - Genome - NCBI (nih.gov) |
| *P. pini* | GWHBHEB00000000 |
| *P. pinifolia* | *Phytophthora pinifolia* (ID 16813) - Genome - NCBI (nih.gov) |
| *P. pisi* | *Phytophthora pisi* (ID 33819) - Genome - NCBI (nih.gov) |
| *P. plurivora* | *Phytophthora plurivora* (ID 56075) - Genome - NCBI (nih.gov) |
| *P. pluvialis* | *Phytophthora pluvialis* (ID 40647) - Genome - NCBI (nih.gov) |
| *P. pseudosyringae* | *Phytophthora pseudosyringae* (ID 88880) - Genome - NCBI (nih.gov) |
| *P. quercina* | *Phytophthora quercina* (ID 95289) - Genome - NCBI (nih.gov) |
| *P. quininea* | *Phytophthora quininea* (ID 88774) - Genome - NCBI (nih.gov) |
| *P. rubi* | *Phytophthora rubi* (ID 31930) - Genome - NCBI (nih.gov) |
| *P. versiformis* | *Phytophthora versiformis* (ID 95293) - Genome - NCBI (nih.gov) |
| *Phytopythium vexans* | *Phytopythium vexans* (ID 35737) - Genome - NCBI (nih.gov) |
| *Pythium aphanidermatum* | *Pythium aphanidermatum* (ID 14200) - Genome - NCBI (nih.gov) |
| *Py. arrhenomanes* | *Pythium arrhenomanes* (ID 14201) - Genome - NCBI (nih.gov) |
| *Py. brassicum* | *Pythium brassicum* (ID 83859) - Genome - NCBI (nih.gov) |
| *Py. guiyangense* | *Pythium guiyangense* (ID 73910) - Genome - NCBI (nih.gov) |
| *Py. insidiosum* | *Pythium insidiosum* (ID 34156) - Genome - NCBI (nih.gov) |
| *Py. myriotylum* | *Pythium myriotylum* (ID 108473) - Genome - NCBI (nih.gov) |
| *Py. oligandrum* | *Pythium oligandrum* (ID 43712) - Genome - NCBI (nih.gov) |
| *Py. periplocum* | *Pythium periplocum* (ID 51249) - Genome - NCBI (nih.gov) |
| *Fusarium brachygibbosum* | *Fusarium brachygibbosum* (ID 103429) - Genome - NCBI (nih.gov) |
| *F. gerlachii* | *Fusarium gerlachii* (ID 35436) - Genome - NCBI (nih.gov) |
| *F. oxysporum* | *Fusarium oxysporum* (ID 707) - Genome - NCBI (nih.gov) |
| *F. redolens* | *Fusarium redolens* (ID 95840) - Genome - NCBI (nih.gov) |
| *F. solani* | *Fusarium solani* (ID 12360) - Genome - NCBI (nih.gov) |
|  |  |
