# Supplementary material for: Rapid detection of Phytophthora cinnamomi based on a new target gene Pcinn13739
Source: Front Cell Infect Microbiol. 2022 Aug 25;12:923700. doi: 10.3389/fcimb.2022.923700 (PMC9452884; doi:10.3389/fcimb.2022.923700)
Supplement: Supplementary Table 2 — Ten loci were randomly selected for screening by conventional PCR assays from more than 1,000 Phytophthora cinnamomi–specific putative genes. [file Table_2.docx]

**Supplementary Table S2** Ten loci were randomly selected for screening by conventional PCR assays from over 1000 *Phytophthora cinnamomi*-specific putative genes.

| **Gene ID** | **Scaffold #** | **Nucletide position** | **DNA sequence** | **Primers and probe** |
| --- | --- | --- | --- | --- |
| *Pcinn13739* | 119 | 84897-85418 | AACGCTGCTACCACCACTGGAGTTGCATCCCCGACCGCAGCTTCAACAACAGGAGCTGCCGCGACGGGCACCACCGGAGCTGCCGTGACAGACACCACTGGCGCGTCAACGGCGGGTGCTTCTACTGAGTCTTCATCAACTGGTGCGACCGGTGCAGCCACCACCAGTGGGTCTTCCACGGGATCTGTCGCCGCCGGAGACACTTCTTCGGCAACCAACAGCATGGATGGTTCCAGCGCGACCATGGCGGGTTCGTCATCCAGTCCCACAATCGATTCGTCGTCGGCAACGGATTCGTCATCCACAGGCACGACTGCAACCTCGAGCACGTCGGGCACCTCGAAAAAGAAATCGATGTCGGGATCGGCCTCCGCGTCGGGCTCCAGTGGTGCGTCGCAAGTGTCCGCGACACTTGGCGCCGCGTCGGCTGCCGTGGTCGCGGTTGCTGCGTACTTCCTG | *Pcinn13739*-F:GCGGGTGCTTCTACTGAGTC *Pcinn13739*-R:TGCCTGTGGATGACGAATCC *Pcinn13739*-nest-F:CCGTGACAGACACCACTGGCG *Pcinn13739*-nest-R:TGCCCGACGTGCTCGAGGTTG *Pcinn13739*-RPA-F:TCAACGGCGGGTGCTTCTACTGAGTCTTCA *Pcinn13739*-RPA-R:Biotin-CAGTCGTGCCTGTGGATGACGAATCCGTTGCCGA *Pcinn13739*-Probe:FAM-GATCTGTCGCCGCCGGAGACACTTCTT-THF-GGCAACCAACAGCATGGATG-C3 |
| *Pcinn16994* | 187 | 26291-26906 | ATGGGCCTGGATAGTGGTGCTGAGTTAGATGAGCAGACGCTTTATGGTGGTGATAGTGACCTCATCGACGACCGCTTTGGCGACAGTGCTTCGGAAGGCGAGCTCTCTGCCTGGAGTGACACGCTTCGGAAGAAGAATGAGCAGACGCTTTATGGTGGTGATAGTGACCTCATCGACGACCGCTTTGGCGACAGTGCTTCGGAAGGCGAGCTCTCTGCCTGGAGTGACACGCTTCGGAAGAAGAGTTCGTTTGATAGCGCGCGTGAAGTGATGGTGGTACTGGGATCTGCGTCGAGGAAATTCAACGCCAACATTCAGGCTTTGCCTCCATGA | *Pcinn16994*-F: CCTGGATAGTGGTGCTGAGTT *Pcinn16994*-R: CAGGCAGAGAGCTCGCCTTCC |
| *Pcinn18321* | 226 | 67199-67694 | ATGGAGAATATGGTCGTGCGCCTGCTGAGCCCGTTCAGAACTACGGCGGACGGCCGCAAGACGGGTGTCTTCGGTGAGGGCCGCGTCGAGGTCGTCCTGCGACTGATCCAGCTGGCGTCGGGTACGCTGGGTCTCGGCTTCGAGGGAAACAACGCGGGCTTGGGCGGCATCGCGAGCAGATCGGATCGGACTGAGGGCTCCGCGAGCCTGGCGCAAGTCGGCTTCCAGACGATCGACATCTTGCTGAAGAGAAGCCCTAGTCAGAGCCCCGGGATGGGGGACGATCTGTGTTGGGGAGGTCGATCGGGCGGCGGATTCGGTCGCCGTCAGATCTGCTTGGACCCGATCTAG | *Pcinn18321*-F: CGCCTGCTGAGCCCGTTCAGA *Pcinn18321*-R: CGTCTGGAAGCCGACTTGCGC |
| *Pcinn1025* | 3 | 768180-768434 | ATGGCGGACGATGGCAGTGTTCAGCTGGTCAAGCTGAGCGTGATGATGAGTGGTGGTCTGATGGTTGTGGTGATGATGGTCGGGCAGTGGAGTCAGCGTGTGACGACAGCGAAGTTGATGGCGATGTGCGGCACCGAGGAGGTCTCTGTGGCGGTGATTATGATGAGGGGCTGCATGTGA | *Pcinn1025*-F: CGGACGATGGCAGTGTTCAGC *Pcinn1025*-F: TCATCATAATCACCGCCACAG |
| *Pcinn10079* | 68 | 288914-289735 | ATGAGTACGACCAAGCTGAAGACCGCCATTGCAACAGAGGTGGTGGTGGAGGTGGTGGAAACACGCCCCGATGGTCAACGCCGTGTTGACCTTCATCTCGTGTCGCTCCACGACCAGGTCACGGTCGAGGAGTACCTCCAGAGCGCGGAAGTTGCCGCTCTGTACCATCGTGTCCCGGCTGTGGCGGTCTCCCAGACCGCCTTGAACGTGCCGGTCGGGCTGTAG | *Pcinn10079*-F: CGACCAAGCTGAAGACCGCCA *Pcinn10079*-R: CCGGCACGTTCAAGGCGGTCT |
| *Pcinn137495* | 213 | 48423-48632 | AATAGCTTGGTGCCCCACTTGTTAGGCTTTGCCTTCATGTACACGCGCAACTTGTTGAAGCTGCTGCGGCTCGCCAGCACGGCTTCGTCGAAGGAGAGCTCGGCTGGGGGCACGTATCCCTCGTGGAATGTACGCTGCAACACTTCTACCACCTTTCTGATCTTCCAGGTCCGGTCCGTCAGTACACGTGGGTCGTCATTATCATTGAAA | *Pcinn137495*-F: CTTGGTGCCCCACTTGTTAG *Pcinn137495*-R: CGTGTACTGACGGACCGGAC |
| *Pcinn14424* | 130 | 96859-97190 | ATGAGTGAATCGACGGTGATGGAGATGGCGACCCTAGCACCACACGCGACGAGTAACATCGACATCACGTACATCACCAACGAGCGCGACATTGACGTCGAGGGCGGTGACGGAGACATCTCCTGCAGTAGCAGCACCGAACACCAGAGCAACGACCACGGAGACGCGAACGATAATACGAGATTCAACCACGACAAGATCGGCGTCGGCGAGGAGACG | *Pcinn14424*-F: TCGACGGTGATGGAGATGGC *Pcinn14424*-R: CCTCGCCGACGCCGATCTTG |
| *Pcinn11754* | 89 | 153769-154155 | ATGGCTGCTGGAAAGAAGAGGAAGTGGGCCACAAAGAAGACAAGGCAAGCGAGAGGATCGTCCTCAAGTGCGAGTGGTGGTGCGGGAGATGAGCTGGAAGGGGAGGATGACCTCGCTGGAAACCCTGACGACAATACGCCGTCGACTCGCCGGGAACGAACTGACGAGTACACTCCCGGTGGGCCCGACCGCGCACGCCCCTCGCGCCCACGTGCCAATGACGACAGCGACGATGACCCTGACAACGAGAGCAGTGGTGACGACAGCGACGGCGGCAGTGATGATGGTGATGATGGAAGCAGCAACGCAGGGGATAGCCTCGGGGGCTCGACGCTCTGGTTGCACGCCCGCCGGAAGGGGATCGCCCAAGCCGCAAAGAGATTGTAA | *Pcinn11754*-F: ACAAGGCAAGCGAGAGGATC *Pcinn11754*-R: CACCATCATCACTGCCGCCG |
| *Pcinn1601* | 5 | 616038-616478 | ATGCAGGTCGTTACGTTTCAAGCAGCGCGATTCCTGCTTTGCCAGCCCGGAAAGGGACAGCGGAGCCGCTCGCGTGCGAAGCAGTCACCGCGAATTTTGGCACGCAGAGACAAGCGCCGCAAGCGGCCAAGCAGCCATGGCGCCTTCGTCAGCGTGGCGCGCGAAGTAACCGCGCCGTCACCGCCGCGCAAAACCCCATCGGCTCCGATCGGAGCCGAGCTCACAGACGTCCGAGCGGAACGCCGGACAGCGCTAGGAGCCACAAGCGCGCTCGTATACGGGGCCAGCATGCCCGGTCCTTGTGCGGCGGACGCGTACGTGGGAGTTCCGGTCATGAGCGCGGGCATTACTCGACGCGAGGCGCAGCGCCGCAAGCAAACACGGAGAGACCGTGGAAGGGCGTCGCCCAGCTGGCGGCCTCCTAGCCGCCCGGTTCCTTGA | *Pcinn1601*-F: GACAGCGGAGCCGCTCGCGT *Pcinn1601*-R: CGTCCGCCGCACAAGGACCG |
| *Pcinn17552* | 202 | 274-869 | ATGGGTGTCTTTGGTGAGGGCCGCGTCGAGGTCGTCCTGCGACTGATCCAGCTGGCGTCGGGTACGCTGGGTCTCGGCTTCGAGGGAAACAACGCGGCTTGGGCGGCATCGCGAGCAGATCGGATCGGACTGAGGGCTCCGCGAGCCTGGCGCAAGTCGGGTACTCGAGGTGACGTCCAGATCGTGGCAGGTGAGATCCAGGTCATGTTCTTGAACCGTGATCTGCAATCGCAGGTCAGCGATCTCCGATGTGGCCAGATCGAGGTCGTGTTCCGTGCTCCGGTTGGAGGACAACGCCCCTTCCAGTTCCTGTCGGGCGCCAGGAGGGCGATCCGGCGTGGTCCATTGCGGGAACTTCGAGTGGAGTGCCGAGAGGCGACGGAGTGTGTCGATCGAGGCGAAGCTGGAGGCGATCCCTCGCCCTCAGATGGGGTGGAGGGTCGAGCAAGCGATCCGCCGGACTGA | *Pcinn17552*-F: CCGCGTCGAGGTCGTCCTGCG *Pcinn17552*-R: CGATTGCAGATCACGGTTCAA |
